# Sustained delivery of MMP-9 siRNA via thermosensitive hydrogel accelerates diabetic wound healing

**DOI:** 10.1186/s12951-021-00869-6

**Published:** 2021-05-05

**Authors:** Biyun Lan, Liming Zhang, Liqun Yang, Junfeng Wu, Na Li, Chenglin Pan, Xiaoyi Wang, Lexiang Zeng, Li Yan, Chuan Yang, Meng Ren

**Affiliations:** 1 Department of Endocrinology, Sun Yat-Sen Memorial Hospital, Sun Yat-Sen University, Guangzhou, 510120 P. R. China; 2 Department of Endocrinology, Guangzhou First People’s Hospital, School of Medicine, South China University of Technology, Guangzhou, 510180 P. R. China; 3 DSAPM Lab and PCFM Lab, School of Materials Science and Engineering, Sun Yat-sen University, Guangzhou, 510275 P. R. China; 4 Department of Polymer and Material Science, School of Chemistry, Key Laboratory for Polymeric Composite and Functional Materials of Ministry of Education, Guangdong Provincial Key Laboratory for High Performance Polymer-based Composites, Sun Yat-Sen University, Guangzhou, 510275 P. R. China; 5 Department of Pediatric Surgery, Sun Yat-Sen Memorial Hospital, Sun Yat-Sen University, Guangzhou, 510120 P. R. China

**Keywords:** Diabetic wound healing, Matrix metalloproteinase-9 (MMP-9), Thermosensitive hydrogel, siRNA delivery

## Abstract

Excessive expression of matrix metalloproteinase 9 (MMP-9) impedes healing of diabetic chronic wounds, thus wound dressing that could effectively inhibit the expression of MMP-9 offers significant clinical translation for diabetic wound healing. Herein, a hybrid hydrogel dressing was developed for localized and sustained delivery of MMP-9 siRNA (siMMP-9). siMMP-9 was complexed with Gly-TETA (GT), the GT/siMMP9 complex was then loaded into a thermosensitive hydrogel based on Pluronic F-127 (PF) and methylcellulose (MC). *In vitro*, this hybrid hydrogel dressing exhibited negligible cytotoxicity, prolonged the release of GT/siMMP-9 for up to 7 days, and significantly reduced MMP-9 expression. *In vivo* assessment in diabetic rats demonstrated that hydrogel provided localized and sustained delivery via the thermosensitive controlled release of entrapped GT/siMMP-9 into wound tissues for 7 days, resulting in dramatic MMP-9 silencing which significantly improved diabetic wound closure. This hybrid hydrogel dressing exhibited excellent biocompatibility, with no observed systemic toxicity in rats. Taken together, the hybrid hydrogel dressing may constitute an effective and biocompatible means of enhancing diabetic wound healing through effective silencing of the MMP-9 gene, and this hydrogel delivery system also offers a platform for *in vivo* delivery of siRNA for the treatment of other diseases.

**Supplementary Information:**

The online version contains supplementary material available at 10.1186/s12951-021-00869-6.

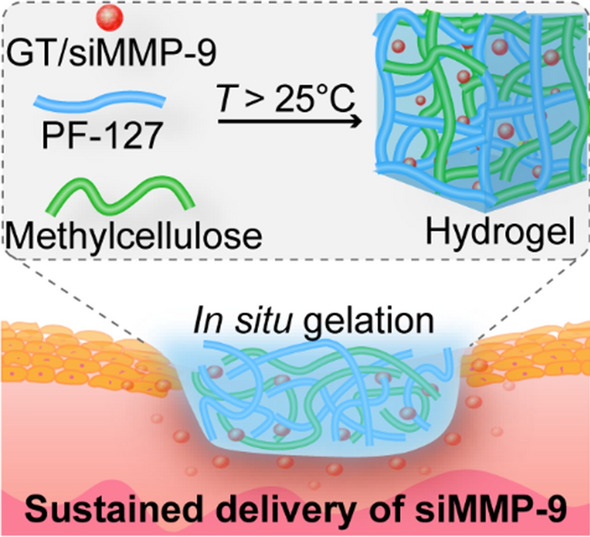

## Introduction

Diabetic chronic wounds are a growing problem worldwide as it is one of the most serious complications in diabetic patients [1]. With limited regenerating capacity after injury, diabetic wounds adversely affect the quality of life (QOL) of patients; and the 5 year-survival is only 29 –50 % [2, 3]. In the United States alone, more than 110,000 diabetes-related amputations occur per year, account for about 75 % of nontraumatic lower extremity amputations; and the diabetes-related amputations is increasing in young (age 18–44 years) groups [4]. The annual cost associated with diabetic chronic wounds ranged from $9 to $13 billion; in addition to the costs associated with diabetes itself. Diabetic chronic wounds continue to be a heavy burden on public health system and patients [5]. Therefore, new cost-effective and safe strategies of wound care management are needed for these hard-to-heal diabetic chronic wounds.

Wound healing of the skin requires a well-orchestrated integration of various physiologic factors and biological events, especially the balance between the deposition of extracellular matrix (ECM) and their remodeling [6]. Matrix metalloproteinases (MMPs) are a zinc-dependent endopeptidase family involved in tissue remodeling. MMP-9 levels are known to be elevated in various diseases, including chronic non-healing wounds, myocardial infarction, stroke and cancers [7–10]. In diabetic chronic wounds, excessive activation of MMP-9 leads to the breakdown of local ECM, and cell migration is impaired without the scaffold provided by ECM. As a result, epithelial closure of the wound is impeded and thus the risk of infection is increased [7, 11]. Since excessive MMP-9 has been demonstrated to be a major pathogenic contributor in delayed diabetic wound healing, down-regulation of MMP-9 expression in local diabetic wounds could be a promising method of facilitating the healing of diabetic wounds.

Our previous studies reported that topical administration of microRNA (miR-129 and -335) in diabetic rats inhibited MMP-9 protein expression, thus promoted diabetic wound healing [12]. However, the lack of specificity and biocompatibility has hampered the clinical potentials of miR-129 and -335 [13]. Alternatively, MMP inhibitors, such as marimastat or rebimastat, may be developed as a therapeutic agent [14]. However, more than 50 clinical trials on MMP inhibitors so far have failed. One of the main reason of failure was the low specificity of these inhibitors, which resulted in off-target MMP inhibition and led to unacceptable side-effects, such as musculoskeletal pain and inflammation [15, 16].

Small interfering RNA (siRNA) holds great potential for therapy due to its precise mode of action and ability to effectively silence targeted gene expression. siRNA-based therapies have been investigated for a range of diseases, including cancer, neurological disorders, cardiovascular diseases and other genetic disorders [17–19]. The U.S. FDA has approved the first siRNA, Onpattro (Patisiran), as a new class of drugs for the treatment of peripheral nerve disease [20]. Our previous studies reported that intradermal injection of β-CD-(D_3_)_7_/siMMP-9 nanoparticles (NP) around the wound bed of diabetic rats significantly reduced the expression of MMP-9 and improved wound healing [21, 22]. However, repeated injections were required to sustain long-term silencing of MMP-9, which might be detrimental to the wound healing process. Moreover, some β-CD-(D_3_)_7_/siMMP-9 NPs escaped into the blood circulation after injection and accumulated in the liver [22].

Polysaccharides-based vectors have shown great potentials for siRNA delivery owing to the outstanding biocompatibility and biodegradability. Glycogen is one of the hyperbranched polysaccharides and shows higher transfection efficiency than linear polysaccharides. To confer glycogen the characteristic to possess positive charges, triethylenetetramine (TETA) was used as the modifying agent [23]. Here, Gly-TETA (GT) was used as the siRNA carrier. The therapeutic potential of siRNA was also limited by other substantial delivery barriers, including degradation by endogenous nucleases and insufficient sustained bioactivity in the target tissue [24]. Therefore, rational depots are needed for sustained and localized siRNA release. Thermosensitive hydrogels are three-dimensional networks that can retain large amounts of water and can undergo a transition from liquid precursor solutions to gels in the wounds. The formation of hydrogels *in situ* can easily fill arbitrarily-shaped defects [25, 26]. Moreover, their porous structure serves as a natural scaffold for high drug-loading capacity [27–29]. The potential of Pluronic F-127 (PF) and methylcellulose (MC) for thermosensitive hydrogel application have been studied due to their unique thermoreversible phase-transition property [30–32]. In this study, the thermosensitive hydrogel made of PF and MC (named PM hydrogel hereafter) was used as a depot for the delivery of siMMP-9.

**Scheme 1:**
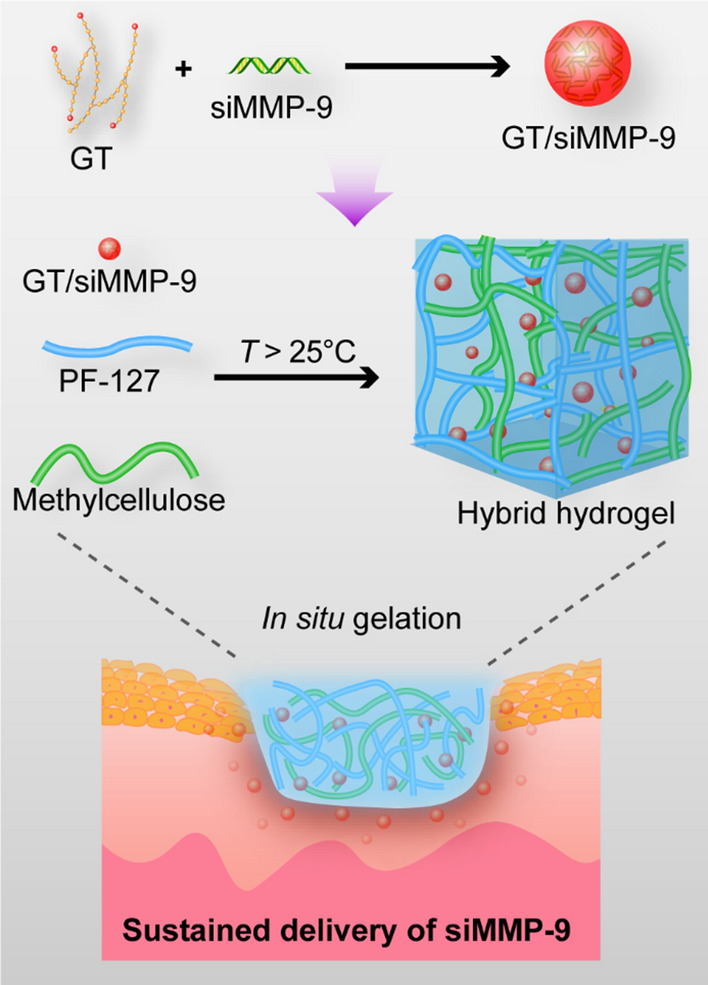
Schematic Illustration of preparation of the hybrid hydrogel dressing. The siMMP-9 is first complexed with GT, then GT/siMMP-9 is mixed completely with PF-127 and methylcellulose. This hybrid hydrogel undergoes a sol-gel transition *in situ* and then deliver sustained siMMP-9 to the diabetic wound area

Herein, we developed a hybrid hydrogel dressing for the localized and prolonged delivery of siMMP-9. The siMMP-9 was first complexed with GT via electrostatic interaction, then GT/siMMP-9 was encapsulated in PM hydrogel. This hybrid hydrogel dressing shows the following features: (i) the thermosensitive hybrid hydrogel could be formed *in situ* and forms a strong interface with arbitrarily-shaped wounds; (ii) localized and sustained delivery of siMMP-9 from hydrogel results in significant MMP-9 silencing, and leads to the acceleration of diabetic wound healing; (iii) no observation of skin allergy or systemic toxicities demonstrates good biocompatibility (Scheme 1).

## Materials and methods

### Materials, cells and animals

All siRNA duplexes were synthesized by GenePharm (Shanghai, China). The sequence of human siMMP-9: 5’-CUA UGG UCC UCG CCC UGA ATT-3’, antisense: 5’-UUC AGG GGC GAC CAU AGT T-3’; the sequence of rat siMMP-9: 5’-GGG CUU AGA UCA UUC UUC ATT-3’, antisense: 5’-UGA AGA AUG AUC UAA GCC CAG-3’; the sequence of negative control siRNA (siRNA): 5’-UUC UCC GAA CGU GUC ACG UTT-3’, antisense: 5’-ACG UGA CAC GUU CGG AGA ATT-3’; siRNA labeled with FAM was abbreviated as siFAM; siRNA labeled with Cy5 was abbreviated as siCy5. Human keratinocyte cells (HaCaT cells, Catalog No. GDC106) were purchased from the China Center for Type Culture Collection (Wuhan, China) and cultured in DMEM (Thermo Fisher, MA, USA) containing 10 % FBS. All cells were maintained under 5 % CO_2_ at 37 °C (Thermo Fisher, MA, USA). Sprague Dawley (SD) rats (6 weeks, male) were purchased from the Guangdong Medical Laboratory Animal Center (Guangdong, China) and maintained under a specific pathogen-free barrier facility at the Laboratory Animal Center of Sun Yat-sen University. All *in vivo* procedures were carried out at the Laboratory Animal Center of Sun Yat-sen University. Protocols described in this study had been approved by the Institutional Animal Care and Use Committee of Sun Yat-sen University (No.00187524 and No.00187525).

### Synthesis and characterization of GT

GT was synthesized by conjugating TETA (Aladdin Biochemical, Shanghai, China) to the hydroxyl groups of glycogen (Sigma-Aldrich, Darmstadt, Germany) using the N,N’-carbonyldiimidazole (CDI) activation method [33]. In brief, glycogen (0.10 g, 0.62 mmol glucose units) was dissolved in 10 mL of anhydrous DMSO, then CDI (5.58 mmol, 0.90 g, Bailingwei Technology, Beijing, China) was added under nitrogen and the mixture was stirred for 1 h. TETA (5.44 g, 37.2 mmol) was subsequently added and reacted at room temperature for 24 h. The solution was dialyzed against distilled water (molecular weight cut-off; MWCO:14,000) for 3 days and lyophilized. Fourier transform infrared (FTIR) spectra was recorded on a Nicolet/Nexus 670 FTIR spectrometer (Thermo Fisher, MA, USA) at a resolution of 4 cm^−1^ by the KBr method. ^1^H nuclear magnetic resonance (NMR) spectra was recorded on a Varian INOVA500NB NMR Spectrometer (Varian, USA) at 500 MHz, using the signal at δ 4.67 ppm for HDO as an internal standard.

### Preparation of the GT/siRNA complex

GT was dissolved in nuclease-free water at 2.15 mg/mL and was then filtered through a Millipore filter (pore size 0.22 μm). Different volumes of GT solutions were mixed with siRNA, the weight ratio of GT to siRNA was varied from 0.5:1 to 30:1. These mixtures were then dispersed in nuclease-free water or opti-MEM (Thermo Fisher, MA, USA) and adjusted to the same final volumes, and the final concentration of siRNA was 0.54 µg/mL. The mixture was incubated for 30 min at room temperature before use. As shown in Additional file 1: Table S1, different siRNA, i.e. siMMP-9, siFAM and siCy5, were used in different experiments. The X in GT/siRNA_X_ represents the weight ratio of GT to siRNA.

### Characterization of the GT/siMMP-9 complex

The zeta potential and size of the complex were determined using ZetaPALS (Brookhaven, USA) at 25 °C with a 90° scattering angle by dynamic light scattering (DLS). The complexation of siMMP-9 to GT was also confirmed by agarose gel electrophoresis. GT/siMMP-9 complexes was suspended in nuclease-free water, 3 µL 6× gel loading buffer was added to each sample and 15 µL of the mixture was loaded onto each well in 4 % agarose gel with 1× GoldView II nuclear staining dye (Solarbio, Beijing, China). Electrophoresis was run in TBE buffer (pH 8.3) at 80 V for 20 min and the gel was observed by G:Box F3 imaging system (Syngene, Cambridge, UK). The morphologies of GT/siMMP-9 complexes were observed on an S-4800 scanning electron microscope (SEM, HI-9056-0003, Hitachi, Japan).

### In vitro cytotoxicity of GT/siMMP-9 complex

#### CCK-8 assay

Cell viability was determined by CCK-8 assay (Dojindo, Japan). HaCaT cells were seeded in 96-well plates (5000 cells/well) in 100 µL of DMEM containing 10 % FBS for 24 h. The medium was then replaced with 100 µL of opti-MEM containing GT/siMMP-9, followed by a 24 h incubation. 20 µL of CCK-8 was added in each well and kept for an additional 2 h. Absorbance at 450 nm was measured using the Tecan Spark 10 M (Tecan, Shanghai, China) spectrophotometer. Cells without treatment were used as the negative control group, and solutions containing DMEM and CCK-8 without cells was used as blanks. Cell viability (%) = (A_450−sample_ − A_450−blank_)/ (A_450−control_ − A_450−blank_) × 100 %.

#### Cell death assay

Cell death was determined by CytoTox96 Nonradioactive Cytotoxicity assay (Promega, WI, USA) based on the calorimetric detection of the released lactate dehydrogenase (LDH). HaCaT cells were seeded in 96-well plates (5000 cells/well) and was incubated with 100 µL of opti-MEM containing GT/siMMP-9 for 24 h. 50 µL of supernatant was mixed with CytoTox96 and was kept for 30 min. Then, 50 µL of stop solution was added to the supernatant. 490 nm absorbance was recorded with a Tecan Spark 10 M spectrophotometer. Cells which were treated with lysis buffer were considered as the positive control group, cells without treatment were used as the negative control group, whereas solutions containing DMEM, CytoTox96 and stop solution without cells were used as the blank group. Cytotoxicity (%) = (A_490−sample_ − A_490−blank_)/(A_490−positive control_ − A_490−blank_) × 100 %.

#### Apoptosis assay

Early (Annexin V+, 7-AAD−) and late (Annexin V+, 7-AAD+) stage apoptosis (Annexin V Apoptosis Detection Kit, eBioscience, MA, USA) was detected by flow cytometry. HaCaT cells were plated in 6-well plates (40,000 cells/well) with 2 mL of DMEM containing 10 % FBS for 24 h. Then, the medium was replaced with opti-MEM containing GT/siMMP-9 and cultured for another 24 h. Cells were harvested, washed twice with PBS, resuspended in 1× Binding Buffer, stained with eFluor 450 conjugated Annexin V and 7-AAD for 15 min at room temperature in the dark, and then analyzed by BD FACSCelesta (BD Biosciences, CA, USA).

### *In vitro* transfection efficiency and lysosomal escape of GT/siRNA

#### FACS analysis

The cellular uptake of the GT/siFAM complex was evaluated by flow cytometry. HaCaT cells were seeded in 6-well plates (40,000 cells/well) with 2 mL of DMEM containing 10 % FBS. After 24 h, the medium was changed into opti-MEM containing GT/siFAM and was further incubated for 4 h. The cells were then rinsed and cultured in fresh medium for 20 h. Cells were collected, washed twice with PBS, resuspended in PBS and subjected to BD FACSCelesta for quantitative analysis. Cells without treatment were considered as the negative control. The efficiency of cellular uptake was represented as the intensity of fluorescein.

#### Confocal microscopy analysis (CLSM)

CLSM was used to observe the cellular uptake. HaCaT cells were seeded in 35 mm dishes with glass bottoms (20,000 cells/well) for 24 h. Then, the cells were incubated with opti-MEM containing GT/siCy5 for 4 h, thereafter the medium was replaced with fresh medium and cultured for another 20 h. Cells were washed three times with PBS, fixed with 4 % paraformaldehyde (PFA) for 15 min, stained with Hoechst 33342 (1 µg/ml) for 2 min, and imaged with a Zeiss LSM800 confocal microscope (Carl Zeiss Meditec AG, Jena, Germany). Cells without treatment were used as the negative control.

#### Lysosomal escape

CLSM was also used to observe the lysosomal escape of siCy5. HaCaT cells were seeded in 35 mm dishes with glass bottoms (20,000 cells/well) for 24 h. Then, the cells were stained with LysoTracker Green (KeyGen, Nanjing, China, 1:1000) for 1 h, incubated with GT/siCy5 for 4 h, rinsed and incubated in fresh medium for another 8 h. The cells were then fixed with 4 % PFA for 15 min, counterstained with Hoechst 33342 (1 µg/ml) for 2 min and observed with the Zeiss LSM800 confocal microscope.

### In vitro MMP-9 silencing of GT/siMMP-9

HaCaT cells were seeded in 6-well plates with 2 mL of DMEM containing 10 % FBS for 24 h. The medium was then replaced with opti-MEM containing GT/siMMP-9, followed by a 4 h incubation. The cells were washed with PBS and cultured in DMEM containing 10 % FBS for another 20 h. Cells without treatment were considered as negative control.

#### Quantitative real‐time polymerase chain reaction (qRT-PCR)

The expression of MMP-9 mRNA was assayed with qRT-PCR. Total RNA was extracted by TRIzol (TAKARA, Japan) according to the manufacturer’s protocols. Reverse transcription of mRNA into cDNA was carried out using the PrimeScript RT Master Mix (TAKARA) according to the manufacturer’s protocols. qRT-PCR was performed on a LightCycler 480II (Roche, Switzerland) with TB Green Premix Ex Taq II (TAKARA) and the primers are shown in Additional file 1: Table S2. Data were analyzed using LightCycler480 software.

#### Western Blot analysis

The expression of MMP-9 protein was detected by Western Blot. Cells were lysed in lysis buffer supplemented with a protease inhibitor cocktail. Protein lysates mixed with loading buffer were heated at 95 °C for 5 min and were then loaded onto 10 % SDS- PAGE gels. The proteins were separated out by electrophoresis and transferred to a polyvinylidene difluoride (PVDF) membrane. Membranes were blocked with 5 % BSA for 1 h at room temperature, incubated with MMP-9 antibody (1:1000, Abcam, Massachusetts, USA) and β-actin antibody (1:5000, Abcam) overnight at 4 °C, then incubated with HRP-conjugated secondary antibody for 1 h at room temperature. Blots were detected by the enhanced chemiluminescence (ECL) system (Millipore, Germany) and imaged with the Mini Chemi610 Imaging System (Sagecreation, Beijing, China).

### Fabrication, optimization and characterization of PM hydrogels

PM hydrogel was made by blending PF with MC. To prepare PM hydrogel at different MC:PF wt. ratio, MC was first dissolved in PBS solution (4 wt%) at 4 °C, then appropriate amounts of PF (12, 16 or 20 wt%) was added and kept for 24 h at 4 °C. PM hydrogels were abbreviated as PM1 (12 wt% PF and 4 wt% MC), PM2 (16 wt% PF and 4 wt% MC), and PM3 (20 wt% PF and 4 wt% MC).

The sol-gel transition of the thermosensitive hydrogel was observed at 4 and 37 °C. Rheological properties of gels were measured using an ARES/RFS rheometer (HAKE Company, TA, USA), the elastic modulus (G′) and viscous modulus (G″) were monitored at 5 % of strain and a frequency of 1 Hz when the temperature was increased from 5 to 40 °C at a heating rate of 2 °C /min. The temperature at the cross point of G′ and G″ was defined as the gelation temperature. Hydrogel morphology was characterized by the S-4800 scanning electron microscope.

### Preparation of PM(GT/siRNA) hydrogel

To prepare PM(GT/siRNA) hydrogels, MC and PF were dissolved completely in PBS solution, then mixed with PBS solution containing GT/siRNA complex to form a gel solution. The final concentration of siRNA was 80 µg/mL.

### Release profiles of GT/siFAM complex from PM hydrogels

The solution of PM(GT/siFAM) hydrogels were loaded onto the upper compartment of the transwell insert (pore size: 8 μm, Corning, NM, USA) in a 24-well plate. Samples were kept at 37 °C for gelation before 1.5 mL of PBS was added into the lower compartment. 100 µL of PBS with released GT/siFAM from hydrogels was taken out at predetermined intervals. The wells were replenished with an equal volume of PBS. The amount of the released siFAM was measured by a Tecan Spark 10 M spectrophotometer (Tecan, Shanghai, China) and calculated according to the standard curve of GT/siFAM. The cumulative release was calculated with the following formula: Cumulative release (%) = (W_released−siFAM_ / W_total−siFAM_) × 100 %, where W_released−siFAM_ represents the weight of released siFAM, and W_total−siFAM_ represents the weight of total siFAM.

### In vitro cytotoxicity of hydrogel-released GT/siMMP-9

PM(GT/siMMP-9) hydrogels were prepared in transwell inserts and immersed in 1.5 mL of opti-MEM as described above, opti-MEM with released GT/siMMP-9 was collected at Day-1, Day-7 and Day-14. HaCaT cells were incubated with the released GT/siMMP-9 for 24 h. CCK-8 was then used to assess the cell viability according to the aforementioned protocol.

### In vitro uptake and gene silencing of hydrogel-released GT/siRNA

PM(GT/siRNA) hydrogel was prepared in transwell inserts and immersed in 1.5 mL of opti-MEM, the released GT/siRNA was collected at Day-1, Day-4 and Day-7. HaCaT cells were incubated with the released GT/siRNA for 4 h and then with fresh medium for 20 h. The cellular uptake of the released GT/siFAM was evaluated by flow cytometry. The uptake of the released GT/siCy5 was observed by CLSM. The MMP-9 silencing efficiency of the released GT/siMMP-9 was assessed by qRT-PCR and Western Blot.

### In vivo wound healing experiments

Rats were used for these experiments. Diabetes was induced by a single intraperitoneal (*i.p.*) injection of freshly dissolved Streptozocin (Sigma-Aldrich, Darmstadt, Germany) at a dosage of 55 mg/kg. After 72 h, blood from the tail vein was collected to examine the blood glucose level with a blood glucose meter (Roche, Switzerland). Rats with a blood glucose reading of over 16.7 mmol/L were considered diabetic.

Four weeks after the injection of Streptozocin, rats were anesthetized with isoflurane (confirmed by toe pinch) and the hair on their backs were shaved. After disinfection with alcohol swabs, full-thickness wounds were created on the dorsal surface using a 10 mm punch biopsy without hurting the underlying muscle. Rats were divided into five groups (n = 6), and the wounds were covered with different dressings as the following: (i) non-diabetic rats treated with PBS, (ii) diabetic rats treated with PBS, (iii) diabetic rats treated with GT/siMMP-9 solution, (iv) diabetic rats treated with PM hydrogel or (v) diabetic rats treated with PM(GT/siMMP-9) hydrogel.

Wounds were then covered with sterile gauze (immersed in PBS before use) and bandage, and the rats were individually caged. A ruler was placed beside each wound to record the wound size and were photographed with a digital camera at Day-0, Day-4 and Day-7 after the procedure. All rats were anesthetized at Day-7 post-wounding, the whole wound tissues with a margin of approximately 4 mm of ambient skin were excised, and then sectioned vertically for H&E staining. The wound closure was then calculated with ImageJ software with the following formula: Wound closure (%) = [(wound area_Day−0_ − wound area_Day−7_) / wound area_Day−0_] × 100 %.

### In vivo evaluation of MMP-9 expression

Excised skin tissues of non-diabetic rats and diabetic rats that were obtained at Day-0 and Day-7 were evaluated for MMP-9 expression by qRT-PCR, Western Blots and Immunohistochemistry (IHC). IHC was performed on formalin-fixed paraffin-embedded skin sections. The slices were heated in an oven at 60 °C for 1 h, washed with xylene and alcohol, blocked with goat serum for 10 min, incubated with MMP-9 antibody for 16 h and peroxidase-labeled polymer for 30 min, then stained with DAB + substrate-chromogen solution and hematoxylin. The whole slides were scanned with a Pannoramic MIDI (3D Histech, Budapest, Hungary), then scored by Quant center software based on the proportion of the positive pixels (dark brown as strong positive, light brown as moderate positive, yellow as weak positive). Histochemistry score (H-SCORE) was calculated with the following formula: H-SCORE = P_weak intensity_×1 + P_mod_

_erate intensity_×2 + P_strong intensity_×3, where P represents the percentage of cells.

### Evaluation of collagen in skin wound

To examine the collagen of wound tissues at Day-7 after wounding, Masson’s trichrome stain were performed on the wound sections. To further evaluate the collagen type I and collagen type III, sections were stained with picrosirius red and viewed under a polarized light microscope (Nikon ECLIPSE, Nikon, Japan). The collagen contents were determined by ImageJ software.

### In vivo toxicity

At Day-7 after wounding, the rats were anesthetized and their major organs including the heart, lung, liver, kidneys and spleen were collected. These were then fixed with 4 % PFA and embedded in paraffin. Tissue sections were stained with H&E and viewed under a Nikon Ni-U optical microscope (Nikon). Blood from the inferior vena cava was collected, and aspartate aminotransferase (AST), alanine aminotransferase (ALT), blood urea nitrogen (BUN) and creatinine (Cr) values of the serum were measured with an automatic biochemical analyzer (Siemens, Germany).

### In vivo biodistribution (BioD)

Diabetic rats were randomly divided into three groups (n = 3), then wounds were created using the protocols mentioned above. Wounds of these rats were then covered with either (i) PBS, (ii) GT/siCy5 solution, or (iii) PM(GT/siCy5) hydrogel. At Day-1, Day-4 and Day-7 after wounding, the rats were imaged using an in vivo imaging system (NightOWL II LB983, Berthold, Germany), the distribution and intensity of fluorescence around the wounds were recorded and measured. At the same time, the fluorescent images of blood from the tail vein and the major organs including the heart, lung, liver, kidneys, spleen and muscle were also recorded. To further observe the uptake of NPs, wound tissues with a margin of approximately 3 mm of surrounding skin were excised at Day-1, Day-4 and Day-7, frozen sectioned on a sliding microtome to a thickness of 8 μm vertically, and then incubated with Hoechst 33342 for 10 min. The skin sections were observed under a Zeiss Axio Observer.A1 fluorescence microscope.

### Statistical analysis

All experiments were performed in triplicate. Data were represented as mean ± standard deviation (SD). Statistical significance was determined by a two-tailed Student’s *t*-test between two groups, and One-way ANOVA between multiple groups. *P* value of 0.05 or less was considered statistically significant.

## Results

### Characterization of GT and GT/siMMP-9 complex

**Fig. 1:**
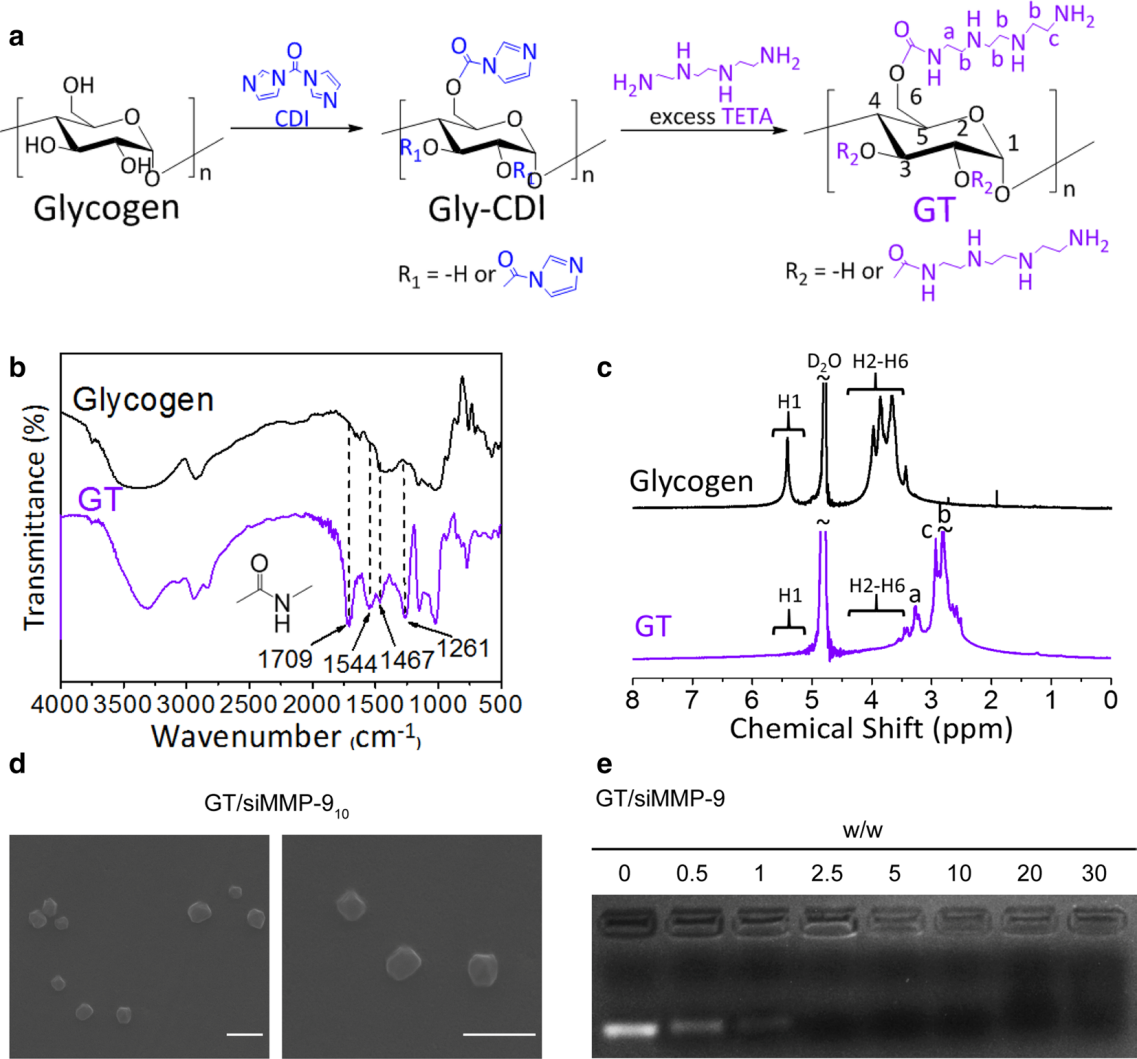
Characterization of GT and GT/siMMP-9 complexes. **a** Chemical synthesis and chemical structure of GT. **b** FTIR spectra of glycogen and GT. **c** ^1^H NMR spectra of glycogen and GT. **d** SEM images of GT/siMMP-9_10_ complexes (scale bars = 500 nm). **e** Agarose gel electrophoresis profiles of GT/siMMP-9_0.5/1/2.5/5/10/20/30_

The successful synthesis of GT was confirmed by FTIR and ^1^H NMR. FTIR spectra showed a characteristic peak at 1,709 cm^−1^ that could be assigned to −CO vibration of the carbamate groups, which indicated that oligoamine residue was conjugated to glycogen with carbamate linkages (Fig. 1b). The proton resonance peaks at 3.2 ppm and 2.8 ppm shown in the ^1^H NMR spectra further confirmed that the oligoamine residues were conjugated with glycogen (Fig. 1c).

The optimum weight ratios of GT to siMMP-9 were explored. According to DLS and gel electrophoresis, for GT/siMMP-9 complexes to possess a positive surface charge, a weight ratio ≥ 2.5 is required (Additional file 1: Table S3 and Fig. 1e). Since the cells membrane possesses negative charge, the cationic nature of the GT/siMMP-9_5/10/20_ favored strong interaction with the cells membrane and hence internalization of the GT/siMMP-9 into cells. Meanwhile, as the weight ratios of GT/siMMP-9 increased, size of this complex gradually reduced. The GT/siMMP-9_5/10/20_ had a diameter of 190–220 nm, which could be internalized through macropinocytosis or clathrin-coated pits, were considered as rational vectors for cellular uptake (Additional file 1: Table S3) [34]. Direct visualization by SEM showed that GT and siMMP-9 self-assemble into approximately spherical NPs (Fig. 1d). Therefore, the weight ratios of GT/siMMP-9 that were selected for further experimentation were 5, 10 and 20.

### In vitro cytotoxicity of GT/siMMP-9_5/10/20_ complex

**Fig. 2:**
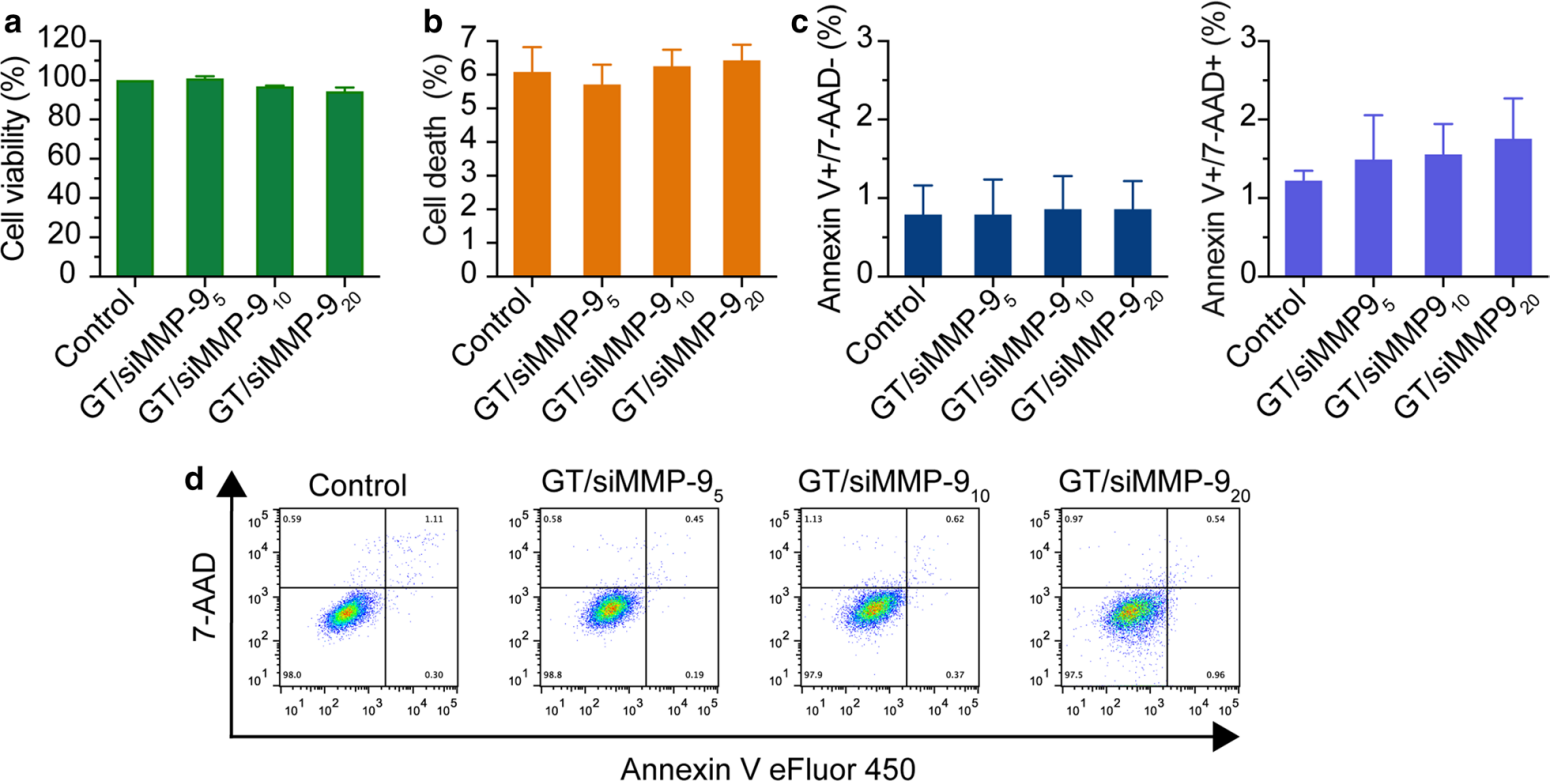
The cytotoxicity of GT/siMMP-9 complexes on HaCaT cells in vitro. **a** Cell viability assessed by CCK-8 assay and **b** Cell death determined by released LDH after 24 h incubation with GT/siMMP-9_5/10/20_ complexes. **c, d** Early (Annexin V+, 7-AAD−) and late (Annexin V+, 7-AAD+) stage apoptosis of cells after 24 h incubation with GT/siMMP-9_5/10/20_

Keratinocyte cells are the major MMP-9 producing cells in skin tissue [12]. The cytotoxicity of GT/siMMP-9_5/10/20_ in HaCaT cells was evaluated. All cells maintained > 95 % viability, with less than 5 % cell death as compared to non-treated cells (Fig. 2a, b). Apoptosis assay indicated that GT/siMMP-9_5/10/20_ could neither induce early nor late stage apoptosis (Fig. 2c, d), indicating the safety of GT/siMMP-9_5/10/20_.

### Cellular uptake and lysosomal escape of GT/siRNA_5/10/20_ complex

**Fig. 3:**
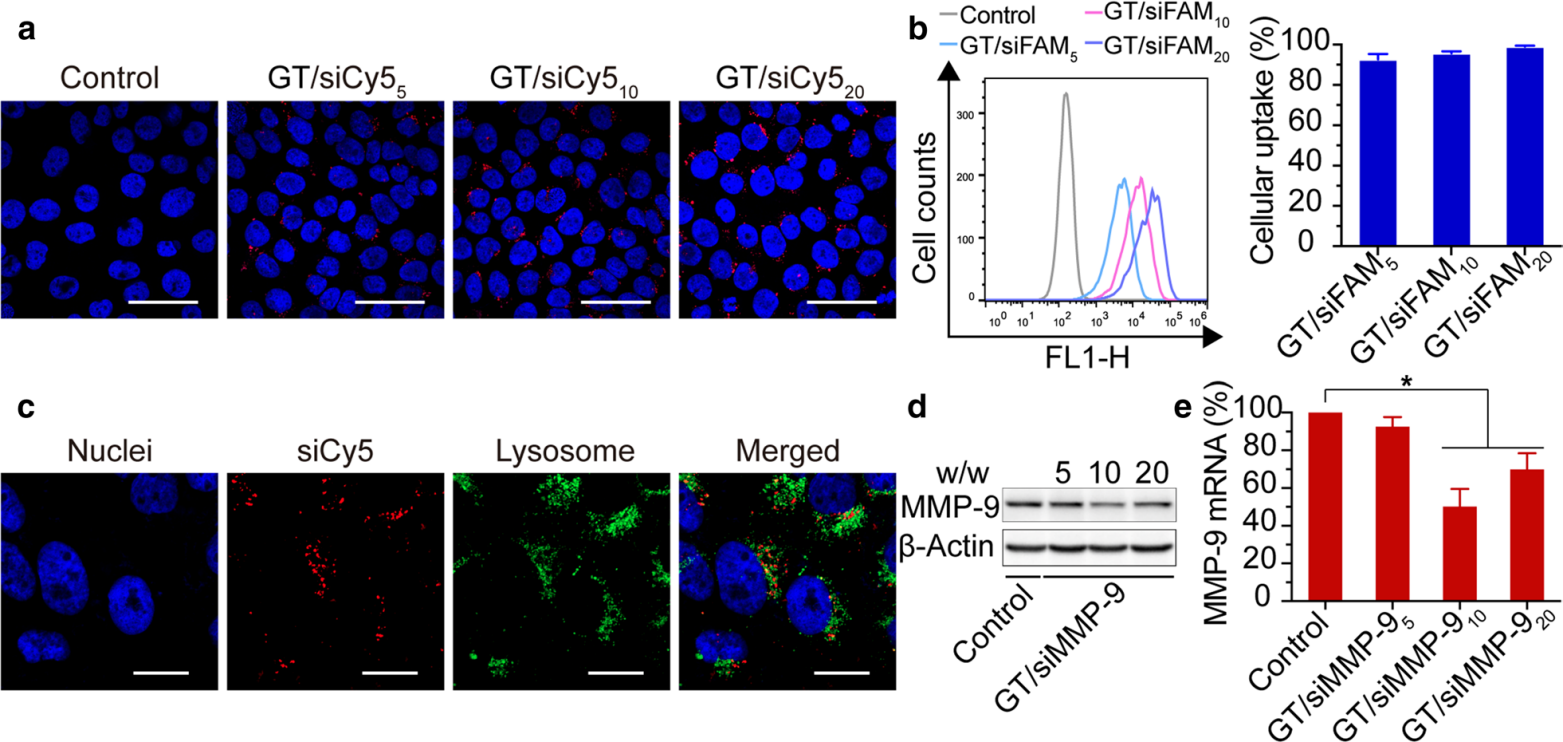
In vitro transfection efficiency and gene knockdown of GT/siRNA_5/10/20_. **a** Images by confocal microscopy of HaCaT cells treated by GT/siCy5_5/10/20_ complexes versus negative control (scale bars = 50 μm). **b** Flow cytometry analysis of transfected HaCaT cells by GT/siFAM_5/10/20_ complexes. **c** Images by confocal microscopy of HaCaT cells incubated with LysoTracker Green and GT/siCy5_10_ complexes for 8 h (scale bars = 20 μm). **d** Western Blot quantification of the cellular level of MMP-9 protein and **e** qRT-PCR quantification of the cellular level of MMP-9 mRNA after treatment with GT/siMMP-9_5/10/20_ complexes versus negative control. *P < 0.05 compared with negative control

The cellular uptake of GT/siRNA_5/10/20_ was assessed by flow cytometry and CLSM. For flow cytometry, siRNA labeled with FAM (siFAM) was used as the indicator of GT/siRNA complex. Results of flow cytometry indicated that GT was able to deliver siFAM into HaCaT cells in a dose-dependent manner, with a cellular uptake efficiency of more than 95 %, and the mean fluorescence intensity was positively correlated with the weight ratio of GT/siFAM (Fig. 3b). For CLSM analysis, siRNA labeled with Cy5 (siCy5) was used as the indicator of GT/siRNA complex. The images of CLSM are in good accordance with the results of flow cytometry (Fig. 3a). Apart from having a high cellular uptake of siRNA, successful release of siRNA from lysosomes into the cytoplasm was also a prerequisite for efficient siRNA-mediated gene silencing [35]. As shown in Fig. 3c, after incubation of 8 h, most of the red fluorescence (GT/siCy5_10_) has separated from the green fluorescence (LysoTracker), indicating that the GT/siCy5_10_ complex can escape from the lysosomes into the cytoplasm.

### In vitro gene silencing of GT/siMMP-9_5/10/20_ complex

Having confirmed the high siRNA uptake via GT, we then examined the gene silence efficacy of GT/siMMP-9_5/10/20_ by qRT-PCR and Western Blot. Results showed that GT/siMMP-9_10/20_ exhibited significant MMP-9 gene knockdown compared to the non-treated control group. And GT/siMMP-9_10_ resulted in greater inhibition on MMP-9 than GT/siMMP-9_20_ (Fig. 3d, e), thus GT/siMMP-9_10_ was selected for future investigation.

### The thermosensitivity of PM hydrogels

**Fig. 4:**
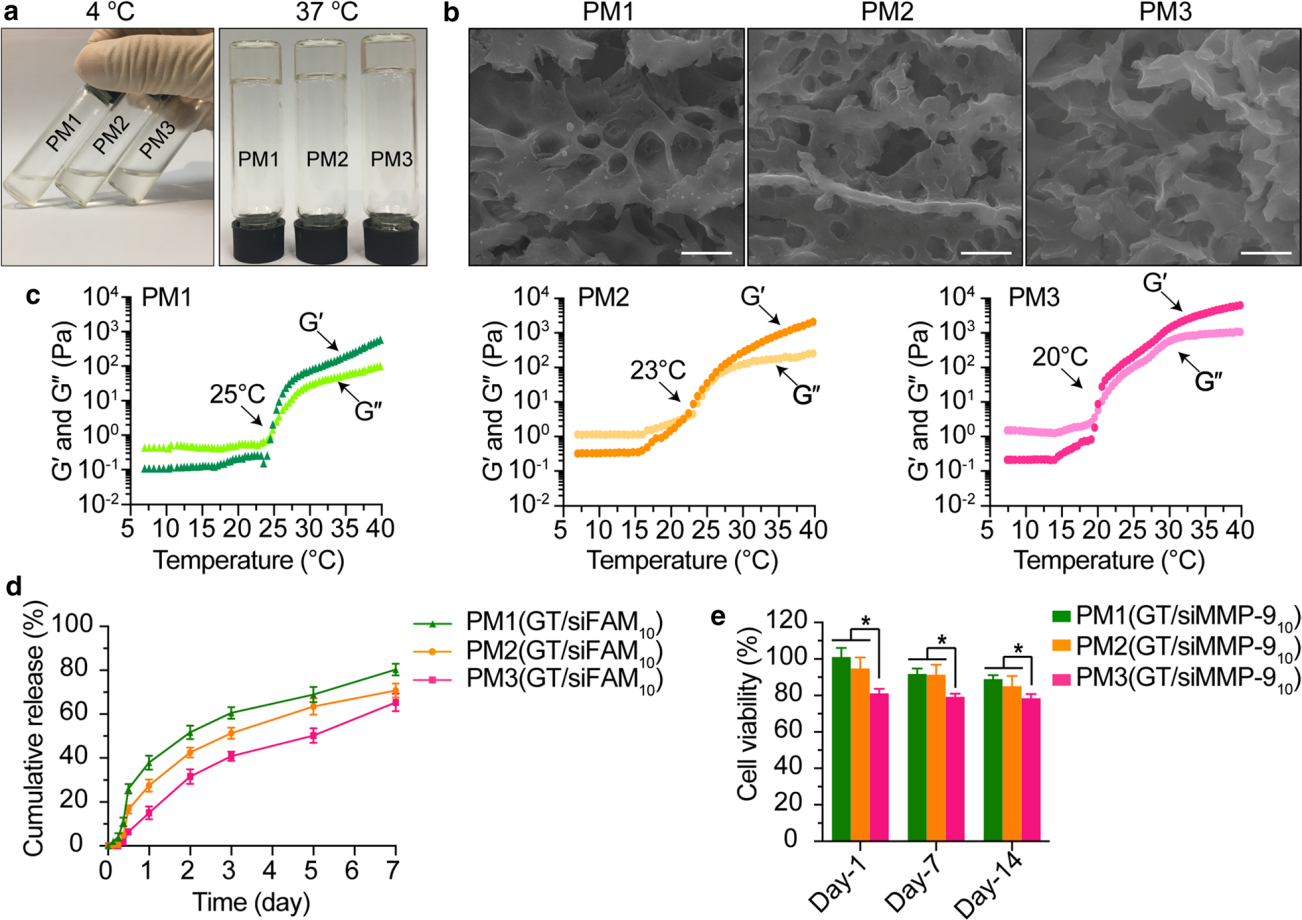
Characterization of PM hydrogels. **a** The sol-gel transition of PM hydrogels with temperature rose from 4 to 37 °C. **b** SEM images of PM hydrogels exhibiting the porous morphology (scale bars = 500 nm). **c** Rheological characterization of PM hydrogels from 5 to 45 °C; the cross point of G′ (dark color) and G″ (light color) was defined as gelation temperature. **d** *In vitro* release profiles of GT/siFAM_10_ from PM hydrogels in PBS at 37 °C. **e** Viability of HaCaT cells assessed by CCK-8 assay after treatment with released GT/siMMP-9_10_ from PM hydrogels at Day-1, Day-7 and Day-14. *P< 0.05

The thermosensitivity of PM hydrogels was confirmed by test tube tilting method and rheology. As shown in Fig. 4a, all PM hydrogels showed thermo-reversible gelation and became gels at 37 °C, indicating that PM hydrogel exists in a gel form at body temperature. The sol-to-gel transition temperatures for PM1, PM2 and PM3 were determined to be 25 °C, 23 and 20 °C as measured by rheology (Fig. 4c). SEM images showed that PM hydrogel was fully filled by an interconnected porous structure (Fig. 4b).

### In vitro release profiles of GT/siFAM_10_ from hydrogels

The *in vitro* release profiles of GT/siFAM_10_ from PM hydrogels were recorded to evaluate the ability of hydrogels as scaffolds for sustained delivery. As shown in Fig. 4d, all PM hydrogels had a controlled release of GT/siFAM_10_ for at least 7 days with a minimal initial burst, additionally about 80 %, 70 and 65 % of the GT/siFAM_10_ were diffused from PM1, PM2 and PM3 gels at Day-7. PM1 showed the highest cumulative release, making it a good candidate for chronic diabetic wound healing.

### In vitro cytotoxicity of PM(GT/siMMP-9_10_) hydrogel

The cytotoxicity test on HaCaT cells was performed with released GT/siMMP-9_10_ from PM hydrogels that were collected at Day-1, Day-7 and Day-14. Compared to PM3 hydrogel, PM1 and PM2 showed much less toxicity toward cells. The viability of cells at Day-14 was greater than 90 % after treatment with PM1, and 85 % after treatment with PM2 (Fig. 4e). PM1 hydrogel had low toxicity towards cells, which allowed its application as potential wound dressings. Thus, PM1 hydrogel was chosen for subsequent experiments.

### Cellular uptake and gene silencing of PM1(GT/siMMP-9_10_) hydrogel

**Fig. 5:**
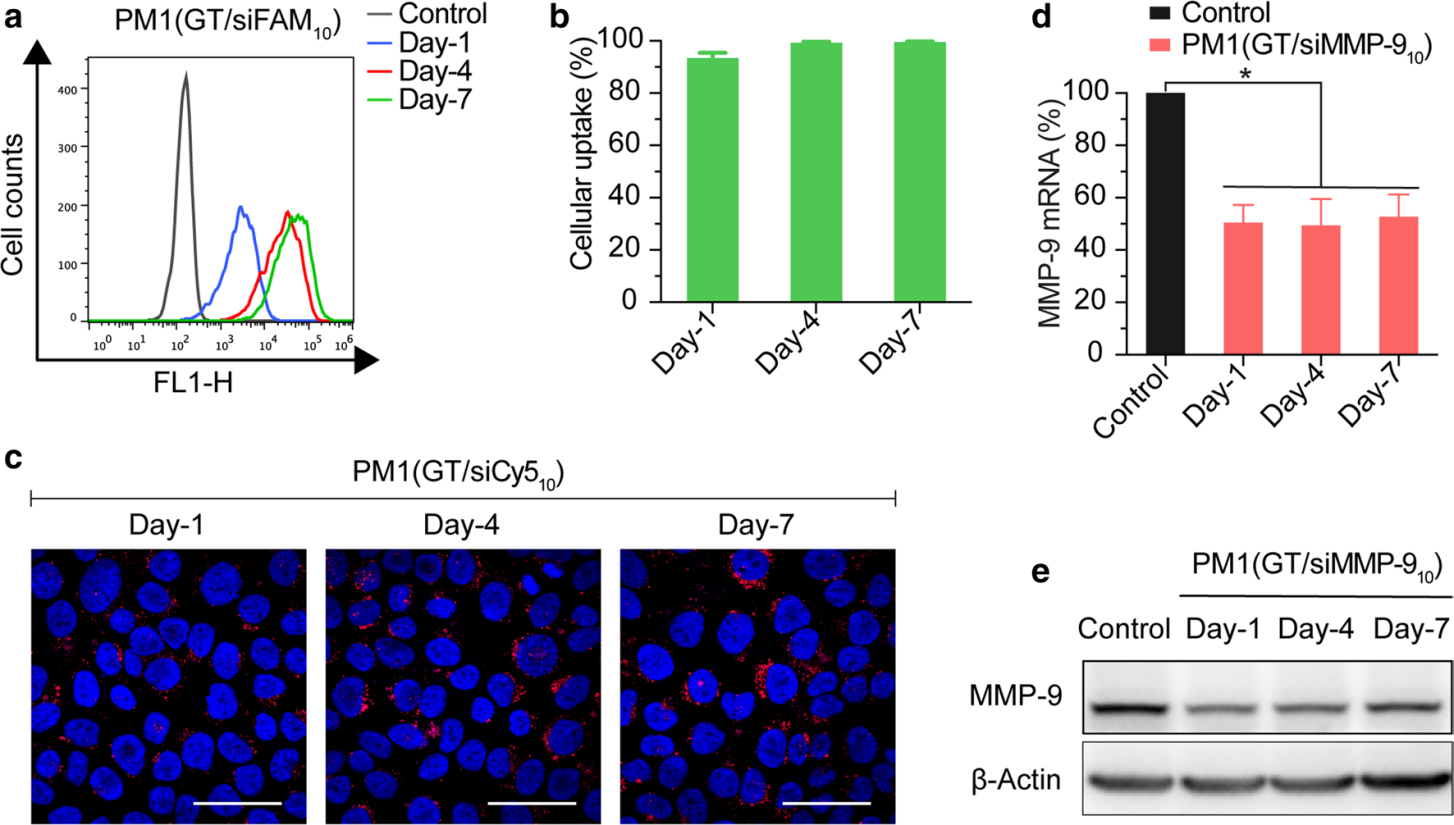
In vitro transfection efficiency of PM1(GT/siRNA_10_) at Day-1, Day-4, Day-7. **a** Flow cytometry analysis of transfected HaCaT cells by released GT/siFAM_10_ from PM1 hydrogel. **b** Cellular uptake after treatments with released GT/siFAM_10_ from PM1 hydrogel. **c** siCy5 uptake of HaCaT cells after treatments with released GT/siCy5_10_ from PM1 hydrogel by CLSM (scale bars = 50 μm). **d** Levels of MMP-9 mRNA in HaCaT cells by qRT-PCR and **e** Levels of MMP-9 protein in HaCaT cells by Western Blot after treatments with released GT/siMMP-9_10_ from PM1 hydrogel. *P< 0.05 compared with negative control

Here, we used siFAM (for flow cytometry) and siCy5 (for CLSM) to determine the uptake of siRNA according to fluorescence intensity. Results of flow cytometry show a successful internalization of GT/siFAM_10_ into HaCaT cells, with a siFAM uptake efficiency of greater than 85 % at Day-1. Fluorescence intensity increased over time as more GT/siFAM_10_ were released into opti-MEM (Fig. 5a, b). Images obtained by CLSM were consistent with flow cytometry analysis, all groups of HaCaT cells showed strong Cy5 fluorescence in cytoplasm (Fig. 5c).

The silencing efficacy of GT/siMMP-9_10_ released from PM1 hydrogel was further validated by Western Blot and qRT-PCR. As shown in Fig. 5d, e, the released GT/siMMP-9_10_ that collected at Day-1, Day-4, and Day-7 can down-regulate levels of MMP-9 mRNA and MMP-9 protein to ~ 50 % when compared to negative control. 7 days after released from PM1 hydrogel, the siMMP-9 still played its role and showed a significant inhibition on MMP-9 expression, indicating the effectiveness of this sustained delivery system for gene silencing.

### In vivo BioD of PM1(GT/siCy5_10_) hydrogel

**Fig. 6:**
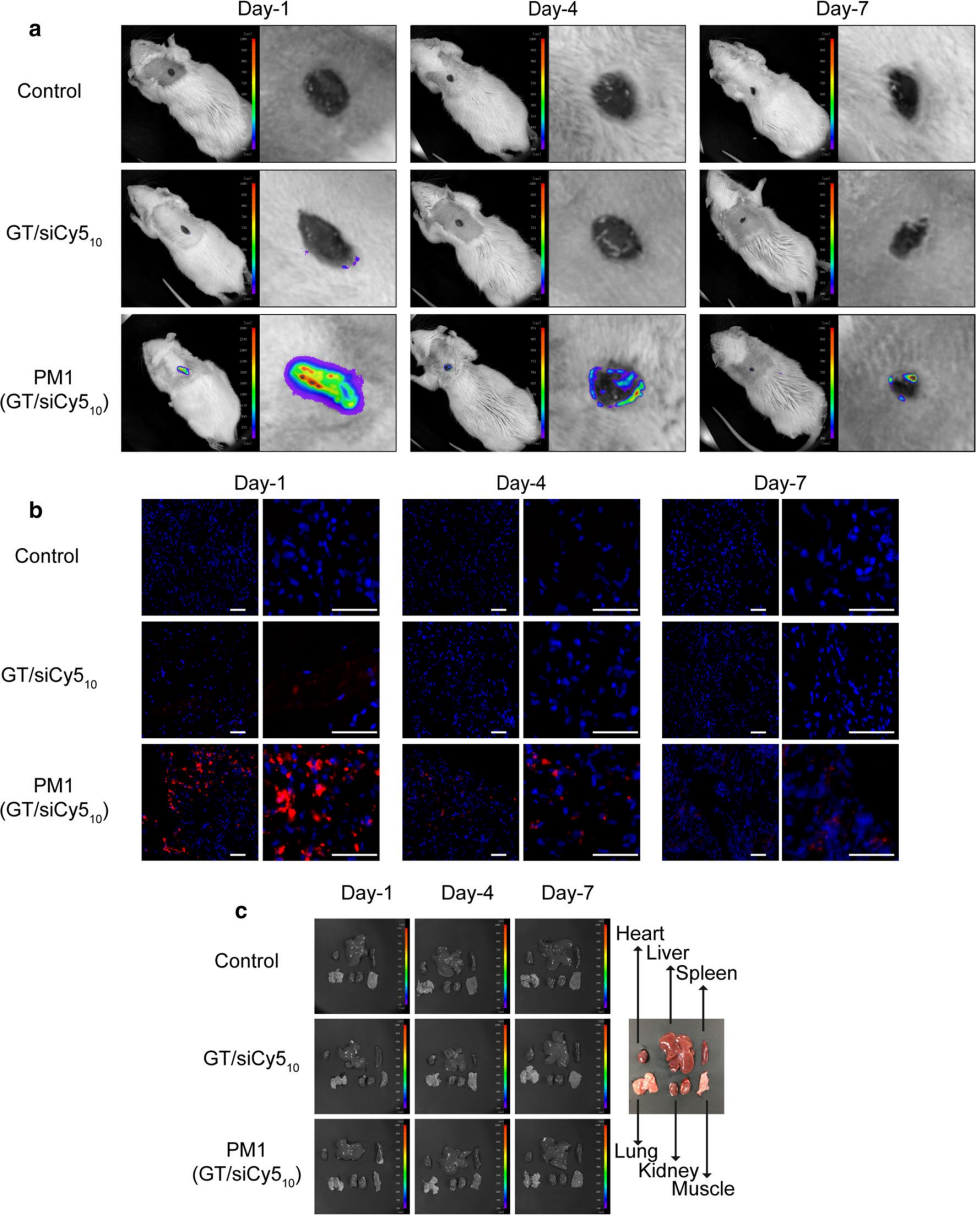
In vivo biodistribution of PM1(GT/siCy5_10_). **a** Fluorescence images of rats at Day-1, Day-4 and Day-7 after treatments of PBS, GT/siCy5_10_ or PM1(GT/siCy5_10_). **b** Fluorescence images of skin sections at Day-1, Day-4 and Day-7 after treatments of PBS, GT/siCy5_10_ or PM1(GT/siCy5_10_) (scale bars = 50 μm). **c** Fluorescence images of main organs of rats sacrificed at Day-1, Day-4 and Day-7

The *in vivo* BioD of siRNA was examined by *in vivo* fluorescence imaging. For this purpose, siCy5 was used for complexation with GT and was encapsulated in PM1 hydrogel. Images acquired at Day-1 after application of PM1(GT/siCy5_10_) hydrogel showed a strong Cy5 fluorescence around the wound, and the fluorescence intensity reduced over time, presumably as a result of the internalization of GT/siCy5_10_ into skin cells, then the siCy5_10_ was incorporated into the RNAi machinery. However, an appreciable fluorescence signal of Cy5 still remained in the wound area 7 days after application of hydrogel (Fig. 6a). In GT/siCy5_10_ solutions, the Cy5 is comparably weaker, and only could be detected one day after administration. Almost no fluorescent signals of Cy5 can be observed at Day-4 and Day-7. To further validate the delivery of siCy5 into skin tissues, wound tissues were excised and sectioned. The fluorescent signals of Cy5 remained detectable at Day-7 day in the group treated with PM1(GT/siCy5_10_) (Fig. 6b). In contrast, in the absence of PM1 hydrogel, rats treated with GT/siCy5_10_ solutions showed a significantly weaker Cy5 fluorescence (only detectable at Day-1). Without a solid scaffold, GT/siCy5_10_ was not able to stay on the wound bed stably, signifying the important role of PM1 hydrogel targeting in the prolonged delivery of siCy5 specifically to skin tissues. To gain insights into the BioD of siCy5, the blood and main organs of rats were harvested and examined. *Ex vivo* imaging results showed no accumulation of siCy5 in blood (Additional file 1: Fig. S3) and these major organs (Fig. 6c). Taken together, these results provide evidence that sustained local delivery of siCy5 to skin tissue can be achieved with the application of PM1(GT/siCy5_10_) hydrogel dressing while limiting non-specific diffusion into the blood circulation, thus avoiding systemic toxicity.

### PM1(GT/siMMP-9_10_) hydrogel reduces MMP-9 expression and promotes wound healing

**Fig. 7:**
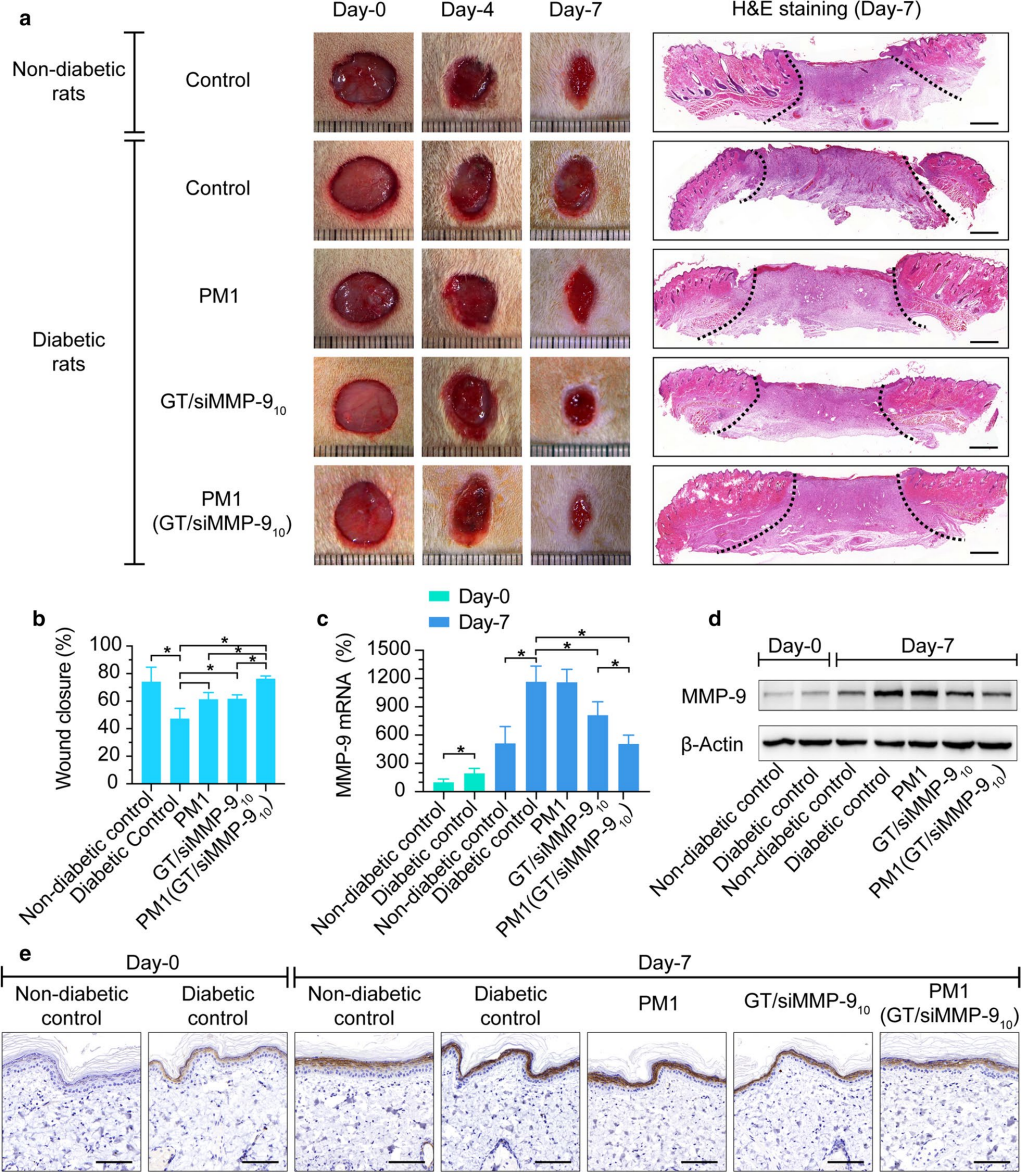
Correlation of MMP-9 expression and wound closure in vivo. **a** Skin wounds after wound creation (Day-0) and at Day-4 and Day-7 after respective treatments of PBS, PM1, GT/siMMP-9_10_ or PM1(GT/siMMP-9_10_) and representative H&E stained sections of Day-7 (scale bars = 1 mm). **b** Wound closure of rats at Day-7. **c** MMP-9 mRNA levels in skin tissues of rats by qRT-PCR at Day-0 and Day-7 after respective treatments. **d** MMP-9 protein levels in skin tissues of rats by Western Blot at Day-0 and Day-7 after respective treatments. **e** Representative images of skin wound tissues by IHC at Day-0 and Day-7 after respective treatments (scale bars = 50 μm). *P < 0.05

With the promising siRNA uptake results described above, we then explored whether PM1(GT/siMMP-9_10_) hydrogel can promote diabetic wound healing. The skin wound model in rats used in this study is a validated and widely used model to evaluate treatments for wound healing [36, 37]. The quantitative analysis of the wound area over time showed that wounds of the non-diabetic group healed dramatically faster (*P* < 0.05) than the diabetic group when both were treated with PBS (Fig. 7a, b). Also, at Day-7 post-wounding, MMP-9 was expressed at basal levels in skin tissues of the non-diabetic group, but was highly expressed in those with diabetes, which was consistent with our previous studies (Fig. 7c–e) [12]. This suggests that the expression and activation of MMP-9 is negatively associated with pathological healing processes and thus the reduction of MMP-9 holds significance for facilitating wound closure.

In comparison, the wound closure of the diabetic group treated with PBS was only ~ 47 % at Day-7, while wound closure of diabetic groups treated with GT/siMMP-9_10_ solution, PM1 hydrogel or PM1(GT/siMMP-9_10_) hydrogel were ~ 61 %, ~ 61 % and ~ 76 % respectively (Fig. 7a, b). GT/siMMP-9_10_ solution, PM1 hydrogel or PM1(GT/siMMP-9_10_) hydrogel significantly improved wound healing, especially PM1(GT/siMMP-9_10_) hydrogel, where a dramatically accelerated wound closure was seen compared to those treated with either GT/siMMP-9_10_ solution or PM1 hydrogel. Meanwhile, the sustained delivery of siMMP-9 from PM1(GT/siMMP-9_10_) hydrogel reduced MMP-9 expression of skin to ~ 43 % as compared to the diabetic group treated with PBS. However, GT/siMMP-9_10_ solution reduced MMP-9 expression to only ~ 70 % (Fig. 7c–e and Additional file 1: Table S4).

**Fig. 8:**
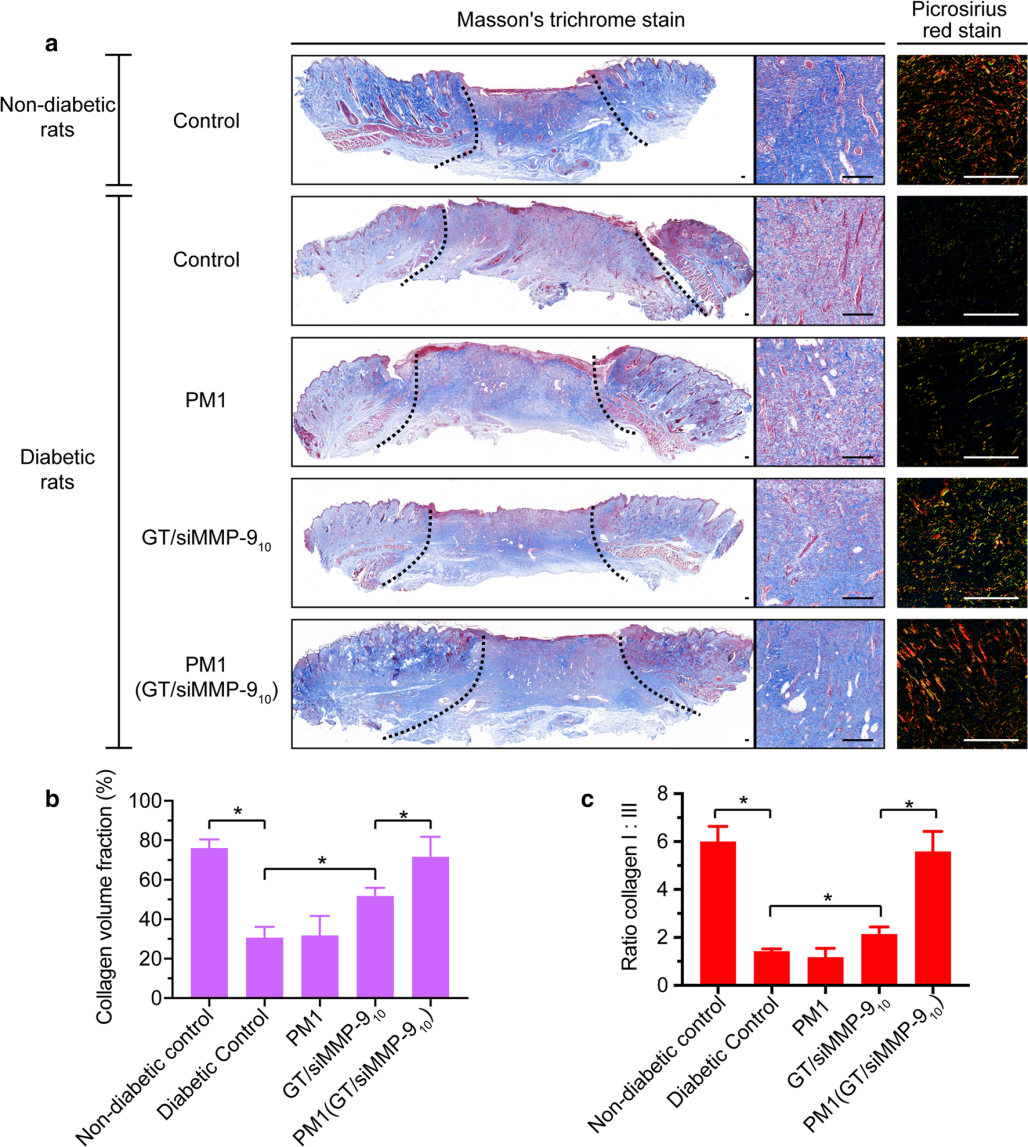
Collagen contents and ratios of collagen type I:III within the wound bed at Day-7. **a** Masson’s trichrome stained and picrosirius red stained wound sections after respective treatments of PBS, PM1, GT/siMMP-9_10_ or PM1(GT/siMMP-9_10_) (scale bars = 100 μm). **b** Collagen volume fraction after respective treatments. **c** Ratios of collagen type I:III after respective treatments. *P < 0.05

As an abundant fibrous protein in the ECM, collagen is also one of the major substrates of MMP-9 within the wound bed [38]. The reduction of MMP-9 is expected to increase the collagen content. Masson’s trichrome stain was used to stain the collagen, which appeared blue. As shown in Fig. 8, the collagen volume fraction (CVF) in the granulation tissue after treatment of PM1(GT/siMMP-9_10_) hydrogel was more than two times of either the diabetic control group or PM1 hydrogel treated group, while the administration of GT/siMMP-9_10_ solution increased only ~ 50 % of CVF. During the process of ECM formation, the thinner collagen type III first appears, and then the thicker collagen type I [38]. To further explore the maturation of granulation tissue, picrosirius red stained wound sections were observed using the polarized light microscope, in which collagen type I appeared red-orange and collagen type III appeared green. As shown in Fig. 8, the ratio of collagen type I:III within wounds of PM1(GT/siMMP-9_10_) hydrogel treated rats was nearly four times of diabetic control rats. The treatment of GT/siMMP-9_10_ solution only increased ~ 60 % of ratio of collagen type I:III when compared with diabetic control rats. These results indicated that the knockdown of MMP-9 via PM1(GT/siMMP-9_10_) hydrogel not only increased the collagen contents within granulation tissue, but also promoted the maturation of granulation tissue, and thus dramatically enhanced diabetic wound healing.

### In vivo biocompatibility of PM1(GT/siMMP-9_10_) hydrogel

To assess the biocompatibility of PM1(GT/siMMP-9_10_) hydrogel, we observed whether it could induce skin allergy or systemic toxicity. In all groups, rats were found to tolerate the application of dressings, as symptoms including erythema, rashes and bumps indicating skin allergy were not noted in the skin surrounding the wounds. In addition, we did not observe liver and kidney toxicity in the rats treated with GT/siMMP-9_10_ solution, PM1 hydrogel or PM1(GT/siMMP-9_10_) hydrogel. Serum AST, ALT, Cr and BUN levels indicated no significant differences in all treated groups compared to negative control (PBS treated group) (Additional file 1: Table S5). Finally, no obvious histopathological abnormalities or lesions of major organs were found from the H&E sections (Additional file 1: Fig. S1). Altogether, these results revealed good biocompatibility of the PM1(GT/siMMP-9_10_) hydrogel.

## Discussion

This study presented a hybrid hydrogel dressing that could achieve the tissue-specific, sustained delivery of siMMP-9 and enhance wound healing in diabetic rats. The PM1(GT/siMMP-9_10_) hydrogel showed rapid gelation in the wound under body temperature, besides demonstrating a prolonged release of siMMP-9 that could act directly at the site of target skin wounds without systemic delivery. It increased the clinical effect of siRNA-based therapy and mitigated the off-target toxicity, thus offering an effective and safe approach for the treatment of diabetic chronic wounds.

To achieve the tissue-specific and sustained delivery of siMMP-9 for diabetic wounds, we developed a hybrid hydrogel dressing that combined the usage of GT/siMMP-9_10_ and thermosensitive hydrogel. In the PM1(GT/siMMP-9_10_) hydrogel, we used the combination of PF and MC as a thermosensitive hydrogel matrix. By varying the percentage of PF and MC to match different desired longevities of GT/siMMP-9 release, the release of siMMP-9 could be readily tuned [39]. Here, the hydrogel hybrid dressing possesses a sustained release of GT/siMMP-9 for about 7 days, which was consistent with the long interval of dressing application of diabetic wounds. Without repeated application, the process of healing could be maximized without interference, enhancing the patients’ QOL. The sustained release of GT/siMMP-9_10_ from the hydrogel exhibited a significant reduction of MMP-9 expression when compared to the solution of GT/siMMP-9_10_, in which the latter required repeated applications. Moreover, in the harsh microenvironment of wounds which exhibited wound fluid containing rich enzymes and RNase, the hydrogel provided a protective environment for the GT/siMMP-9_10_ NPs from being degraded [40]. *In vivo* assessment demonstrated that the application of GT/siMMP-9_10_ solution and PM1 hydrogel both improved wound healing. The GT/siMMP-9_10_ solution could reduce MMP-9 expression and thus in turn increase the amount of collagen within the wound bed [41, 42]. For PM1 hydrogel, hydrogels with high water content was viscoelastic and mechanically similar to biological tissues, which could provide a moist environment for the wound bed. It is well-known that the a moist wound environment effectively promotes cell proliferation and migration besides facilitating re-epithelization [43, 44]. It was noteworthy that PM1(GT/siMMP-9_10_) hydrogel accelerated wound healing much faster than either GT/siMMP-9_10_ solution or PM1 hydrogel, which might be attributed to : (1) the capability of hydrogel acting as suitable scaffold to maximize the therapeutic potential of siMMP-9, and specifically target the anomalous high expression of MMP-9 presented in diabetic wounds, which promotes accumulation of collagen content within the granulation tissue, especially the more mature collagen type I; (2) the sustained delivery of siMMP-9 without repeated application allows minimal interference with the wound healing process; (3) the gelation of PM1(GT/siMMP-9_10_) hydrogel in the wound bed maintains a moist environment surrounding the wounded area.

As an alternative to systemic delivery, local injection of β-CD-(D_3_)_7_/siMMP-9 around the wound bed has demonstrated successful knockdown of MMP-9. However, multiple injection resulted in the accumulation of siMMP-9 in the liver [22]. This indicated that repeated administrations might result in siMMP-9 penetrating the microvessels in the skin, entering into the circulation, and accumulating in the liver due to reticuloendothelial system clearance [45]. Since siRNA could potentially result in thrombocytopenia or peripheral neuropathy after significant systemic exposure [46], this side effect was undesirable for wound dressings. In our hybrid hydrogel dressing, no accumulation of siMMP-9 was observed in the major organs. Therefore, the topical administration of PM1(GT/siMMP-9_10_) hydrogel localized the effects of siMMP-9 and minimized its systemic toxicity.

For wound care that requires the localized and sustained delivery of siMMP-9, other strategies have been explored. It was reported that a siMMP-9 depot on top of a nylon bandage through the use of layer-by-layer assembly could also deliver siMMP-9 directly into a wound bed and promoted wound healing [41]. This bandage-based dressing was easily applied onto the wounds with regular shapes. However, in diabetic wounds, some could be arbitrarily-shaped defects, especially sinus tract wounds. The thermosensitive hydrogel could form *in situ* under body temperature, adapts to the geometry of the wounds and forms a strong interface with skin tissues without destroying natural tissue structure [25, 26, 47]. Therefore, our hybrid hydrogel dressing can be administered in a minimally invasive manner.

When developing wound dressings, the ‘keep it simple, stupid (KISS)’ principal is highly recommended to promote clinical translation. To be widely accepted by the public, the following requirements are needed, such as effectiveness, safety, reproducibility and the ability to upscale production [48]. For diabetic wound treatment, other new dressings have been investigated. Adipose-derived stem cells or exosomes from adipose-derived mesenchymal stem cells was encapsulated into hydrogels, and they remarkably accelerated diabetic wound closure *in vivo *[49, 50]. Despite these promising results, wound dressings using cells or cell components were challenged by the accessibility to human tissue and the isolation/expansion of appropriate amount for clinical applications. The hybrid hydrogel dressing here possesses the advantages of low cost, ease of preparation and could be readily tuned. Additionally, as an effective and safe approach to alter gene expression, the hybrid hydrogel is capable of delivering any siRNA or other therapeutics with structurally similar nucleic acids such as miRNAs to skin wounds. The strategy is also not limited to applications for diabetic chronic wounds, it can be applicable for a myriad of clinical disorder with altered localized protein expression in specific tissue microenvironments, including cancer, neurological disorders and cardiovascular diseases.

## Conclusions

To summarize, we successfully developed a hybrid hydrogel dressing made of PF and MC for the localized and sustained release of siMMP-9 to treat diabetic chronic wounds. The hybrid hydrogel dressing could be formed *in situ* due to its thermosensitive characteristic. It not only provided a moist environment, but also reduced the expression of MMP-9 in local diabetic wounds, and thus accelerated wound healing. This study provides evidence for our hybrid hydrogel dressing as an efficient and safe way for diabetic chronic wounds, and the hybrid hydrogel also shows potential for treating other diseases where pathogenic proteins are over-expressed in the microenvironment.

## Supplementary Information

**Additional file 1: Fig. S1.** In vitro degradation of PM(GT/siMMP-9_10_) hydrogel in PBS at 37 °C.** Fig. S2** (**a**) The measurement of tissue-adhesive ability. (**b**) Adhesive strength of PM(GT/siMMP-9_10_) hydrogel, ethyl cyanoacrylate or PBS. *P < 0.05. **Fig. S3.** Fluorescence images of blood of rats collected at Day-1, Day-4 and Day-7 after treatments of PBS, GT/siCy5_10_ or PM1(GT/siCy5_10_). **Fig. S4.** Histological section of the major organs after respective treatments (from top to bottom, PBS in non-diabetic rats, PBS in diabetic rats, PM1, GT/siMMP-9_10_ or PM1(GT/siMMP-9_10_) (scale bars = 100 μm). **Table S1.** Different siRNA used in the experiments. ** Table S2.** Primer sequence for qRT-PCR.** Table S3.** DLS of GT/siMMP-9 complexes at different weight ratio.** Table S4.** H-SCORE of MMP-9 in rat skin.** Table S5.** Parameters of liver function and kidney function of rats.

## Data Availability

All data generated and analyzed during this research are included in this published article.
