# Supplementary material for: Sustained delivery of MMP-9 siRNA via thermosensitive hydrogel accelerates diabetic wound healing
Source: J Nanobiotechnology. 2021 May 5;19:130. doi: 10.1186/s12951-021-00869-6 (PMC8097905; doi:10.1186/s12951-021-00869-6)
Supplement: Supplementary file 1 — Additional file 1: Fig. S1. In vitro degradation of PM(GT/siMMP-910) hydrogel in PBS at 37 °C. Fig. S2 (a) The measurement of tissue-adhesive ability. (b) Adhesive strength of PM(GT/siMMP-910) hydrogel, ethyl cyanoacrylate or PBS. *P < 0.05. Fig. S3. Fluorescence images of blood of rats collected at Day-1, Day-4 and Day-7 after treatments of PBS, GT/siCy510 or PM1(GT/siCy510). Fig. S4. Histological section of the major organs after respective treatments (from top to bottom, PBS in non-diabetic rats, PBS in diabetic rats, PM1, GT/siMMP-910 or PM1(GT/siMMP-910) (scale bars = 100 μm). Table S1. Different siRNA used in the experiments. Table S2. Primer sequence for qRT-PCR. Table S3. DLS of GT/siMMP-9 complexes at different weight ratio. Table S4. H-SCORE of MMP-9 in rat skin. Table S5. Parameters of liver function and kidney function of rats. [file 12951_2021_869_MOESM1_ESM.docx]

Supplementary Material

**Sustained Delivery of MMP-9 siRNA via Thermosensitive Hydrogel Accelerates Diabetic Wound Healing**

Biyun Lan^1,2†^, Liming Zhang^3†^, Liqun Yang^4†^ , Junfeng Wu^3^, Na Li^1^, Chenglin Pan^3^, Xiaoyi Wang^1^, Lexiang Zeng^5^, Li Yan^1^, Chuan Yang^1^, Meng Ren^1^*

^†^ Biyun Lan, Liming Zhang and Liqun Yang contributed equally to this work.

* Correspondence: renmeng@mail.sysu.edu.cn.

^1^ Department of Endocrinology, Sun Yat-Sen Memorial Hospital, Sun Yat-Sen University, Guangzhou 510120, P. R. China.

^2^ Department of Endocrinology, Guangzhou First People's Hospital, School of Medicine, South China University of Technology, Guangzhou 510180, P. R. China.

^3^ DSAPM Lab and PCFM Lab, School of Materials Science and Engineering, Sun Yat-sen University, Guangzhou 510275, P. R. China.

^4^ Department of Polymer and Material Science, School of Chemistry, Key Laboratory for Polymeric Composite and Functional Materials of Ministry of Education, Guangdong Provincial Key Laboratory for High Performance Polymer-based Composites, Sun Yat-Sen University, Guangzhou 510275, P. R. China.

^5^ Department of Pediatric Surgery, Sun Yat-Sen Memorial Hospital, Sun Yat-Sen University, Guangzhou 510120, P. R. China.

***In vitro* degradation of PM(GT/siMMP-9_10_) hydrogel**

To determine the degradation of PM(GT/siMMP-9_10_) hydrogel, the weight of the 35mm dish was weighed (W_d_). 1 mL of PM(GT/siMMP-9_10_) solution was placed in the dish and was kept at 37 °C for gelation, the initial weight of the dish and hydrogel was weighed accurately (W_i_). 2 mL of PBS was added into the dish and was removed at predetermined intervals, then the hydrogel in dish was weighed (W_t_). The degradation of PM(GT/siMMP-9_10_) hydrogel was calculated with the following formula: Remaining weight of hydrogel = (W_t_ - W_d_) / (W_i_ - W_d_) × 100%.

As shown in Figure S1, the weight loss gradually decreased with increasing amounts of the PF in the hydrogel, and percentages of remaining hydrogel at Day-7 were about 31%, 41%, 50% for PM1(GT/siMMP-9_10_), PM2(GT/siMMP-9_10_) and PM3(GT/siMMP-9_10_).


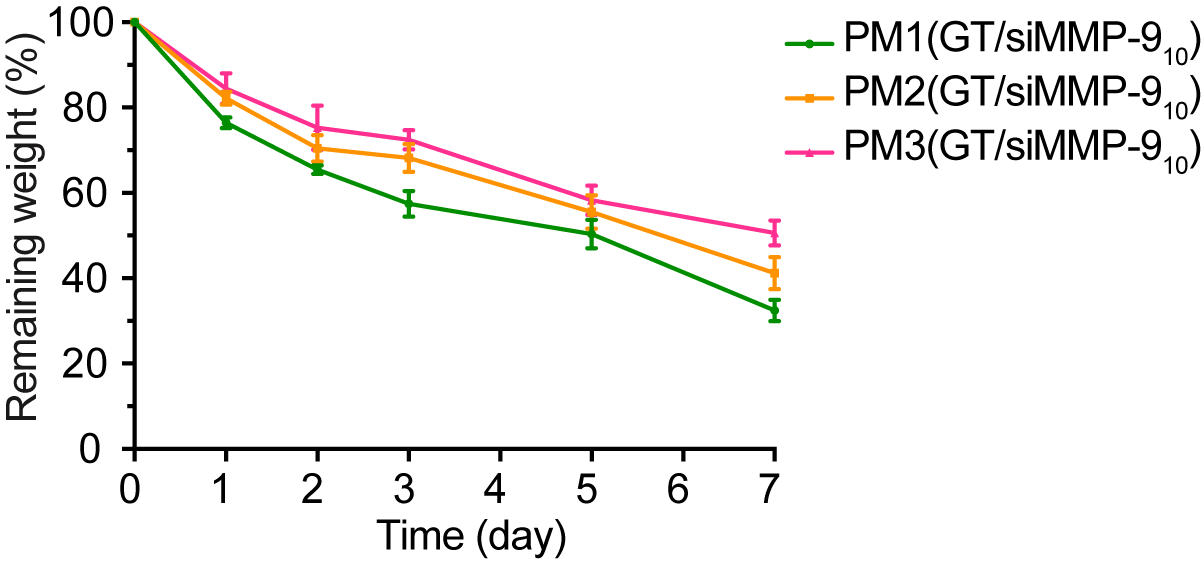


**Fig. S1.** *In vitro* degradation of PM(GT/siMMP-9_10_) hydrogel in PBS at 37 °C.

**Tissue-adhesive abilities of PM(GT/siMMP-9_10_) hydrogels**

The tissue-adhesive abilities of PM(GT/siMMP-9_10_) hydrogels were measured using Tensile Strength Tester (FR-103C, FaRui) with 50 N load cell, and porcine skin was used in this experiment. In brief, the size of each porcine skin’s contact surface were measured and the cross section area was calculated and set as the cross section area parameter in tensile strength tester. Then, 250μL of hydrogels, ethyl cyanoacrylate (as positive control) or PBS (as negative control) was placed between the cross section surface of two pieces of porcine skins, which were pushed together for 1 min. Thereafter the porcine skins were kept at 37℃ for 20 min. The data was collected by Tensile Strength Tester with a loading rate of 0.3 mm/min. The adhesion strength was calculated according to the formula: adhesion strength (kPa) = F/S, where F represents the maximal force before porcine skins seperated and S represents the cross section area.

As shown in Fig. S2 (please see the revised supplementary material), the adhesion strength of PM1(GT/siMMP-9_10_), PM2(GT/siMMP-9_10_) and PM3(GT/siMMP-9_10_) were ~0.8 kPa, ~1.1 kPa, 1.3 kPa, respectively, while the adhesion strength of ethyl cyanoacrylate was ~87.0 kPa. The PM(GT/siMMP-9_10_) hydrogels showed little adhesive property. Since our study aimed to inhibit the expression of MMP-9 of diabetic wound, hydrogel with high adhesion strength was not developed. But we totally agree that adhesion of hydrogel will benefit its further application in wound dressing. In the future, we might continue to work on the multifunctional dressing with the abilities of RNA interference and tissue adhesive.


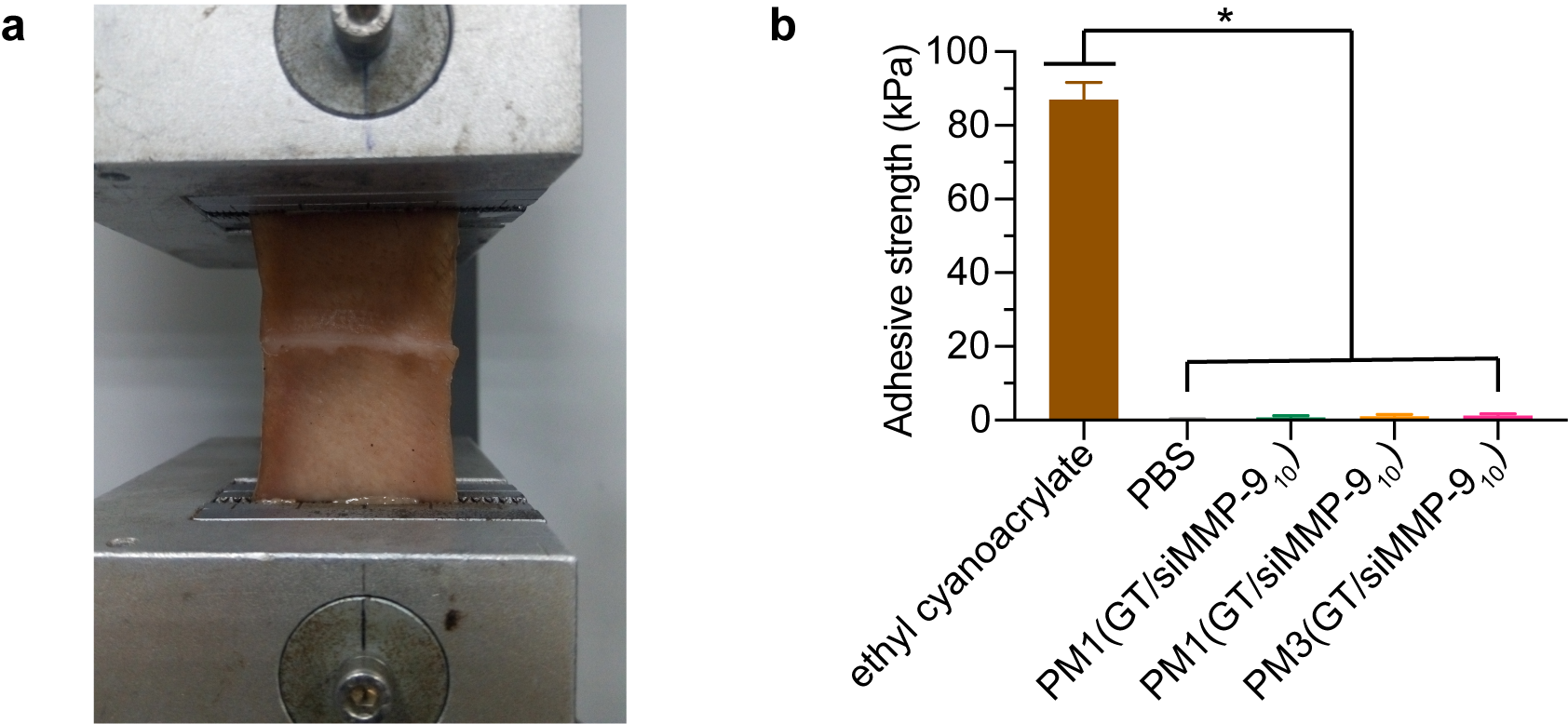


**Fig. S2** (a) The measurement of tissue-adhesive ability. (b)Adhesive strength of PM(GT/siMMP-9_10_) hydrogel, ethyl cyanoacrylate or PBS. **P* ＜ 0.05


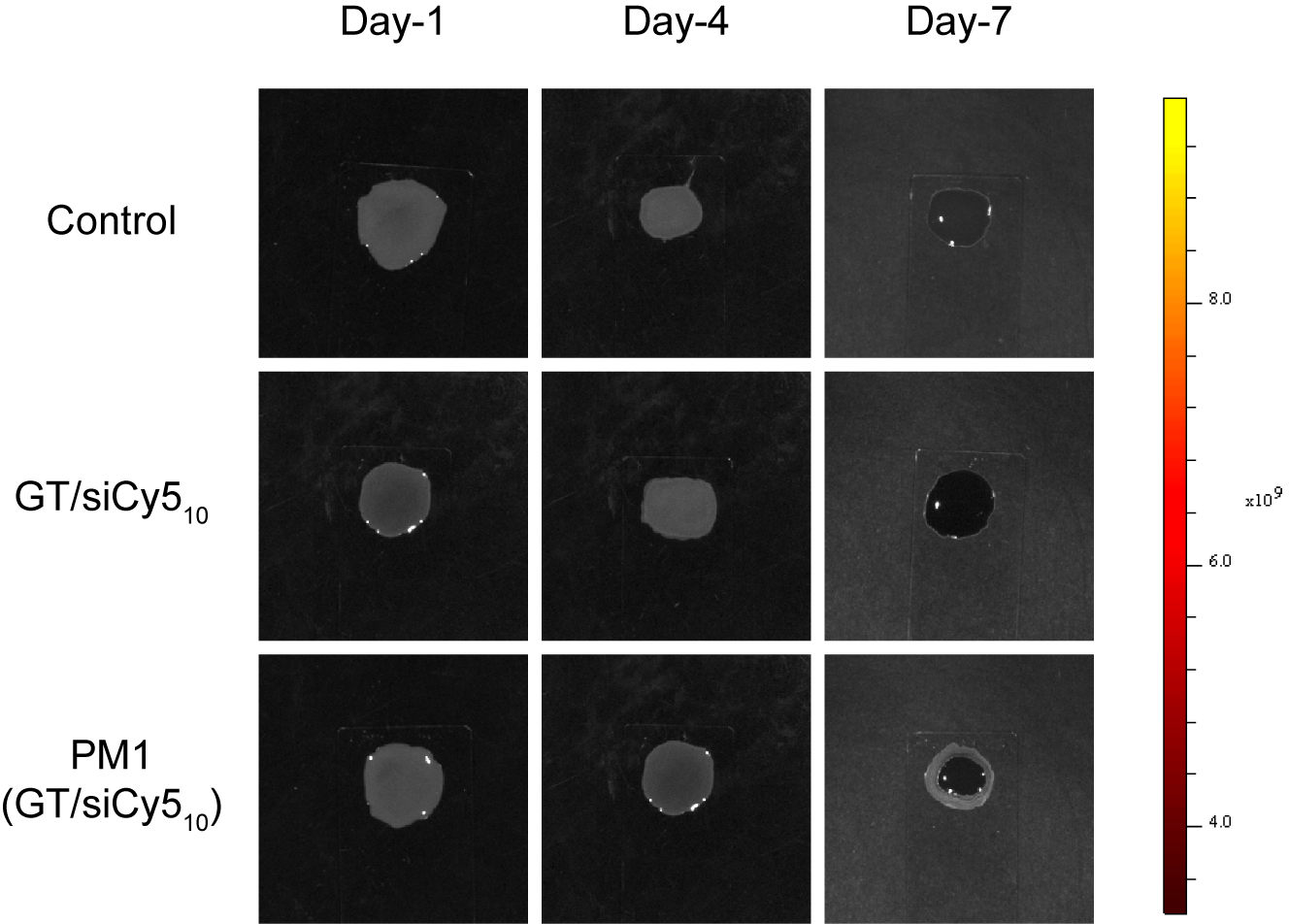


**Fig. S3.** Fluorescence images of blood of rats collected at Day-1, Day-4 and Day-7 after treatments of PBS, GT/siCy5_10_ or PM1(GT/siCy5_10_).


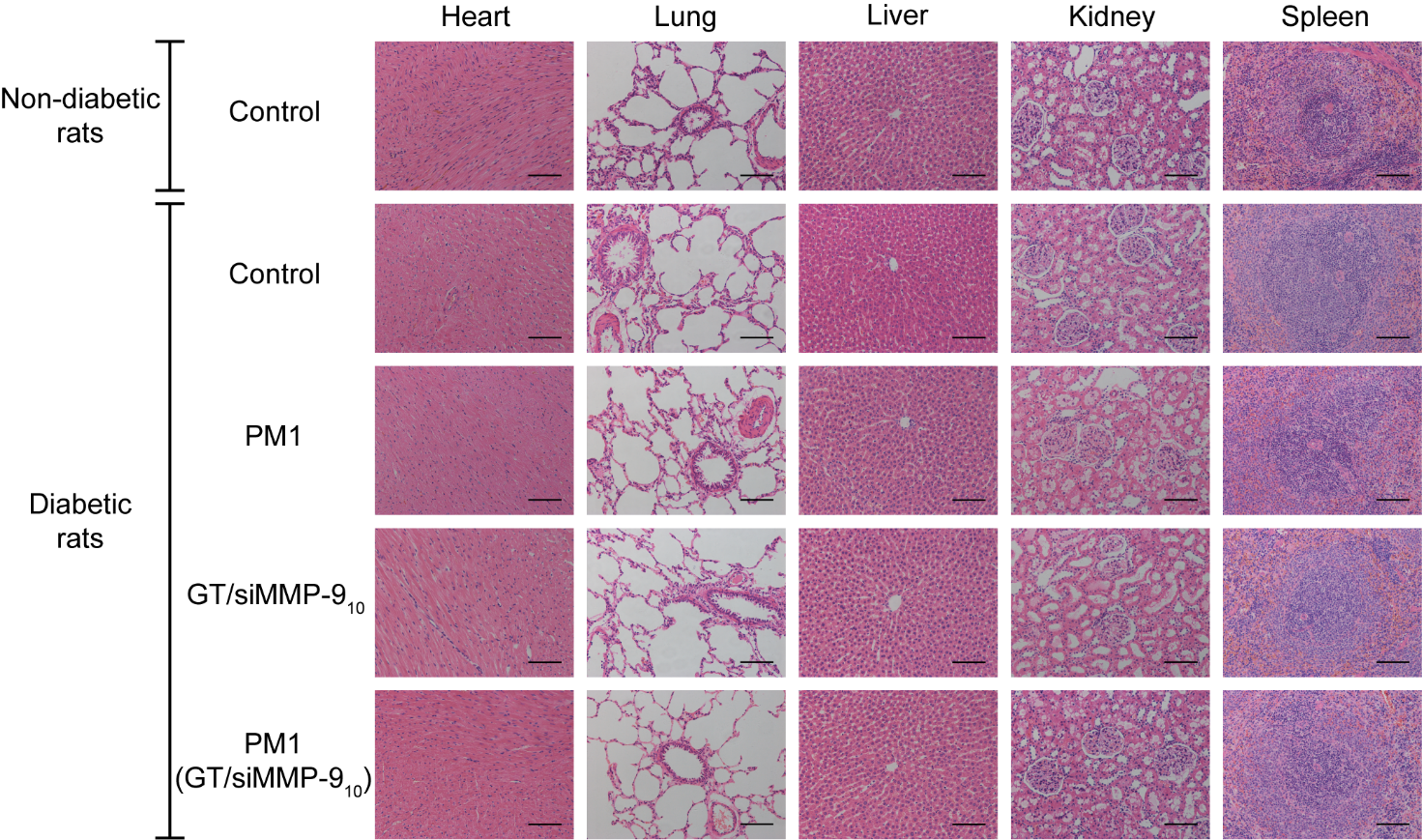


**Fig. S4.** Histological section of the major organs after respective treatments (from top to bottom, PBS in non-diabetic rats, PBS in diabetic rats, PM1, GT/siMMP-9_10_ or PM1(GT/siMMP-9_10_) (scale bars = 100 μm).

**Table S1.** Different siRNA used in the experiments

| siRNA | Objectives | Experiments |
| --- | --- | --- |
| siMMP-9 | Complexation of  GT and siRNA | DLS, agarose gel electrophoresis and SEM |
|  | *In vitro* cytotoxicity | CCK-8, cell death assay and apoptosis assay, |
|  | MMP-9 silencing | qRT-PCR, Western Blot and *in vivo* wound healing |
| siFAM | Cellular uptake | Flow cytometry |
|  | Release profile | Spectrophotometer |
| siCy5 | Cellular uptake | CLSM |
|  | *In vivo* BioD | *In vivo* fluorescence imaging |

**Table S2**. Primer sequence for qRT-PCR

|  | Gene | Forward | Reverse |
| --- | --- | --- | --- |
| Human | MMP-9 | CCCTGGAGACCTGAGAACCAA | CATCTCTGCCACCCGAGTGTA |
| Human | ACTB | TGGAACGGTGAAGGTGACAG | AACAACGCATCTCATATTTGGAA |
| Rat | MMP-9 | CATGCGCTGGGCTTAGATCA | GAGGCCTTGGGTCAGGTTTAGAG |
| Rat | ACTB | GGAGATTACTGCCCTGGCTCCTA | GACTCATCGTACTCCTGCTTGCTG |

**Table S3**. DLS of GT/siMMP-9 complexes at different weight ratio

|  | GT/  siMMP-9_0.5_ | GT/  siMMP-9_1_ | GT/  siMMP-9_2.5_ | GT/  siMMP-9_5_ | GT/  siMMP-9_10_ | GT/  siMMP-9_20_ | GT/  siMMP-9_30_ |
| --- | --- | --- | --- | --- | --- | --- | --- |
| w/w^a)^ | 0.5:1 | 1:1 | 2.5:1 | 5:1 | 10:1 | 20:1 | 30:1 |
| Size (nm) | 357.6±9.6 | 393.5±6.4 | 237.5±5.8 | 217±2.0 | 216±2.0 | 192.9±3.2 | 218.8±3.2 |
| ζ(mV)^b)^ | -5.96±1.45 | -4.37±1.20 | 5.26±2.64 | 7.99±3.78 | 10.9±2.12 | 9.61±1.41 | 11.04±2.16 |

a) w/w: weight ratios of GT to siMMP-9

b) : zeta potential

**Table S4**. H-SCORE of MMP-9 in rat skin

|  | Day-0 | | Day-7 | | | | |
| --- | --- | --- | --- | --- | --- | --- | --- |
| Group | Non-diabetic control | Diabetic control | Non-diabetic control | Diabetic control | PM1 | GT/  siMMP-9_10_ | PM1(GT/  siMMP-9_10_) |
| H-SCORE | 3.02 ±  3.45 | 6.79 ±  4.71 | 17.50 ±  3.11 | 40.50 ±  8.38 | 39.57 ±  9.36 | 27.21 ±  5.68 | 19.54 ±  6.57 |

**Table S5**. Parameters of liver function and kidney function of rats

| Group | Non-diabetic  control | Diabetic  control | PM1 | GT/  siMMP-9_10_ | PM1(GT/  siMMP-9_10_) |
| --- | --- | --- | --- | --- | --- |
| ALT (U/L) | 74.00±29.20 | 85.50±28.54 | 61.67±21.29 | 59.50±5.68 | 64.33±43.99 |
| AST (U/L) | 133.00±53.99 | 167.67±56.81 | 137.50±43.21 | 151.67±29.43 | 126.17±63.50 |
| Cr (μmol/L) | 48.79±6.82 | 57.55±8.90 | 52.76±4.86 | 51.12±5.01 | 51.61±6.36 |
| BUN (mmol/L) | 6.22±0.79 | 9.17±4.37 | 8.18±1.52 | 8.35±1.34 | 7.03±1.84 |
